# Robo4 is constitutively shed by ADAMs from endothelial cells and the shed Robo4 functions to inhibit Slit3-induced angiogenesis

**DOI:** 10.1038/s41598-022-08227-8

**Published:** 2022-03-14

**Authors:** Wenyuan Xiao, Alejandro Pinilla-Baquero, John Faulkner, Xuehong Song, Pradeep Prabhakar, Hong Qiu, Kelley W. Moremen, Andreas Ludwig, Peter J. Dempsey, Parastoo Azadi, Lianchun Wang

**Affiliations:** 1 Department of Molecular Pharmacology & Physiology, Byrd Alzheimer’s Research Institute, University of South Florida, 4001 E. Fletcher Ave., Tampa, FL33613 USA; 2 Complex Carbohydrate Research Center, and Department of Biochemistry and Molecular Biology, University of Georgia, Athens, GA 30602 USA; 3 Institute for Molecular Pharmacology, RWTH Aachen University, Aachen, Germany; 4 Department of Pediatrics, University of Colorado Medical School, Aurora, CO USA

**Keywords:** Cell signalling, Proteolysis, Cell biology

## Abstract

Roundabout 4 (Robo4) is a transmembrane receptor that expresses specifically in endothelial cells. Soluble Robo4 was reported in the human plasma and mouse serum and is inhibitory towards FGF- and VEGF-induced angiogenesis. It remains unknown how soluble Robo4 is generated and if soluble Robo4 regulates additional angiogenic signaling. Here, we report soluble Robo4 is the product of constitutive ectodomain shedding of endothelial cell surface Robo4 by disintegrin metalloproteinases ADAM10 and ADAM17 and acts to inhibit angiogenic Slit3 signaling. Meanwhile, the ligand Slit3 induces cell surface receptor Robo4 endocytosis to shield Robo4 from shedding, showing Slit3 inhibits Robo4 shedding to enhance Robo4 signaling. Our study delineated ADAM10 and ADAM17 are Robo4 sheddases, and ectodomain shedding, including negative regulation by its ligand Slit3, represents a novel control mechanism of Robo4 signaling in angiogenesis.

## Introduction

Roundabout 4 (Robo4) is a transmembrane receptor and belongs to the axon guidance molecule Roundabout (Robo) family, which contains Robo1-4. Robots are most known as the receptor for the family of Slit proteins, including Slit1-3. Robo4 includes an extracellular domain, a transmembrane region, and an intracellular domain and is expressed specifically in endothelial cells^1^. Robo4 mediates intersomitic vessel sprouting in Zebrafish^2^ and Slit3-induced angiogenesis in mice^3^. On the other hand, Robo4 counteracts vascular endothelial cell growth factor (VEGF) signaling to inhibit angiogenesis and stabilize the vasculature^4–6^. These observations highlight that the functions of Robo4 are complex and context-dependent. Currently, it remains unknown how the complex functions of Robo4 are regulated.

Soluble Robo4 was detected in the murine serum^7^ and raises significantly in patient plasma 2 h after cardiac surgery^8^. Meanwhile, soluble Robo4 has been proposed as one of the plasma biomarkers to monitor the clinical course of cerebral cavernous malformations^9^. The functional importance of soluble Robo4 was investigated using the recombinant Robo4 extracellular domain. Recombinant Robo4 extracellular domain inhibits VEGF- and fibroblast growth factor (FGF)-induced angiogenesis in a subcutaneous sponge angiogenesis mouse model^10^ and VEGF-induced retinal hyperpermeability in mice^6^. These studies have advanced our understanding of the potential clinical value and biological functions of soluble Robo4. However, it remains unknown how soluble Robo4 is generated and if soluble Robo4 regulates additional signaling pathways in angiogenesis. Our previous studies showed that Slit3 is a novel angiogenic factor and binds to Robo4, not Robo1, to promote endothelial cell proliferation and migration and induce angiogenesis in vivo^3^. We also delineated that heparan sulfate, a type of highly sulfated polysaccharide and a member of the glycosaminoglycan family, binds Slit3 and functions as a co-receptor to facilitate the angiogenic Slit3-Robo4 signaling in developing diaphragm^11^. It is unknown if soluble Robo4 modulates the Slit3 signaling in angiogenesis.

Here we report that the soluble Robo4 is produced by constitutive shedding endothelial cell surface Robo4 by a disintegrin and metalloproteinase 10 (ADAM10) and ADAM17, and the shed Robo4 (sRobo4) inhibits angiogenic Slit3 signaling in endothelial cells in culture and neovascularization in vivo. Meanwhile, Slit3 shields Robo4 shedding by enhancing the receptor endocytosis, revealing that Slit3 inhibits Robo4 shedding to enhance Robo4 signaling. Our study delineates ADAM10 and ADAM17 are Robo4 sheddases, and ectodomain shedding represents a novel regulatory mechanism of Robo4 signaling.

## Results

### Robo4 is constitutively shed from the endothelial cell surface

**Figure 1:**
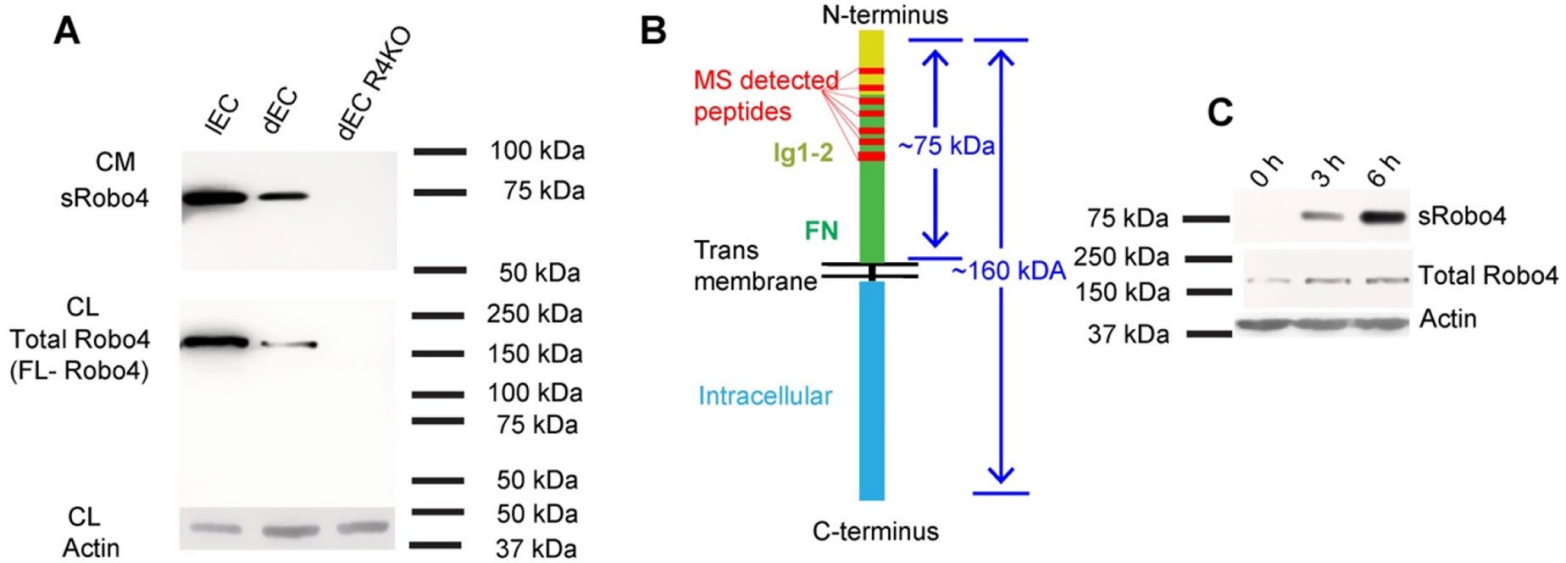
Robo4 ectodomain sheds constitutively. (**A**) sRobo4 was detected in conditioned media (CM) of endothelial cell culture. An antibody specific for Robo4 ectodomain detected a 75 kDa protein band in 6-h serum-free CM and a ~ 160 kDa full-length Robo4 band in the cell lysate (CL) of a wild-type mouse lung EC (lEC) line and a wildtype mouse diaphragm endothelial cell (dEC) line, but not in a *Robo4*-knockout dEC (dEC-R4KO) line. (**B**) sRobo4 was detected in proteomics analysis. 10 peptide sequences of 7 regions (red strips) within the ectodomain of Robo4 were detected in the excised 75 kDa protein band in mass spectrometry (MS) analysis, and the amino acid sequences of the 7 regions were annotated in Supplementary Fig. 1. The amino acid sequences of 10 Robo4 peptides and their corresponding MS information were listed in Supplemental Dataset 1. (**C**) sRobo4 in dEC conditioned medium was measured as in (**A**) and accumulated over time (0, 3, 6 h).

Ectodomain shedding is a regulatory mechanism of cell surface molecules^12,13^. Robo4 was detected in murine serum and human plasma^7,9^, suggesting that Robo4 may be shed from endothelial cells. To directly address this issue, we probed conditioned culture media collected from immortalized murine diaphragm endothelial cells (dEC) and immortalized mouse lung endothelial cells with an antibody specific to the Robo4 extracellular domain. We detected a 75 kDa protein band present only in the conditioned media, not cell lysates (Fig. 1A). The 75 kDa soluble Robo4 was not detected in the conditioned medium of dEC derived from *Robo4* knockout mice (Fig. 1A). Proteomics analysis of the 75 kDa protein band detected peptide sequences within the immunoglobulin (Ig) 1 and Ig2 domains and the fibronectin domains of the ectodomain of Robo4, not within Robo4 transmembrane and intracellular C-terminal domain (Fig. 1B, Supplemental Fig. 1 & Supplementary Data 1), confirming the detected 75 kDa band was sRobo4 in conditioned media. The sRobo4 accumulated over time in culture (Fig. 1C), showing that Robo4 shedding occurs constitutively.

### ADAM10 and ADAM17 are the Robo4 sheddases

**Figure 2:**
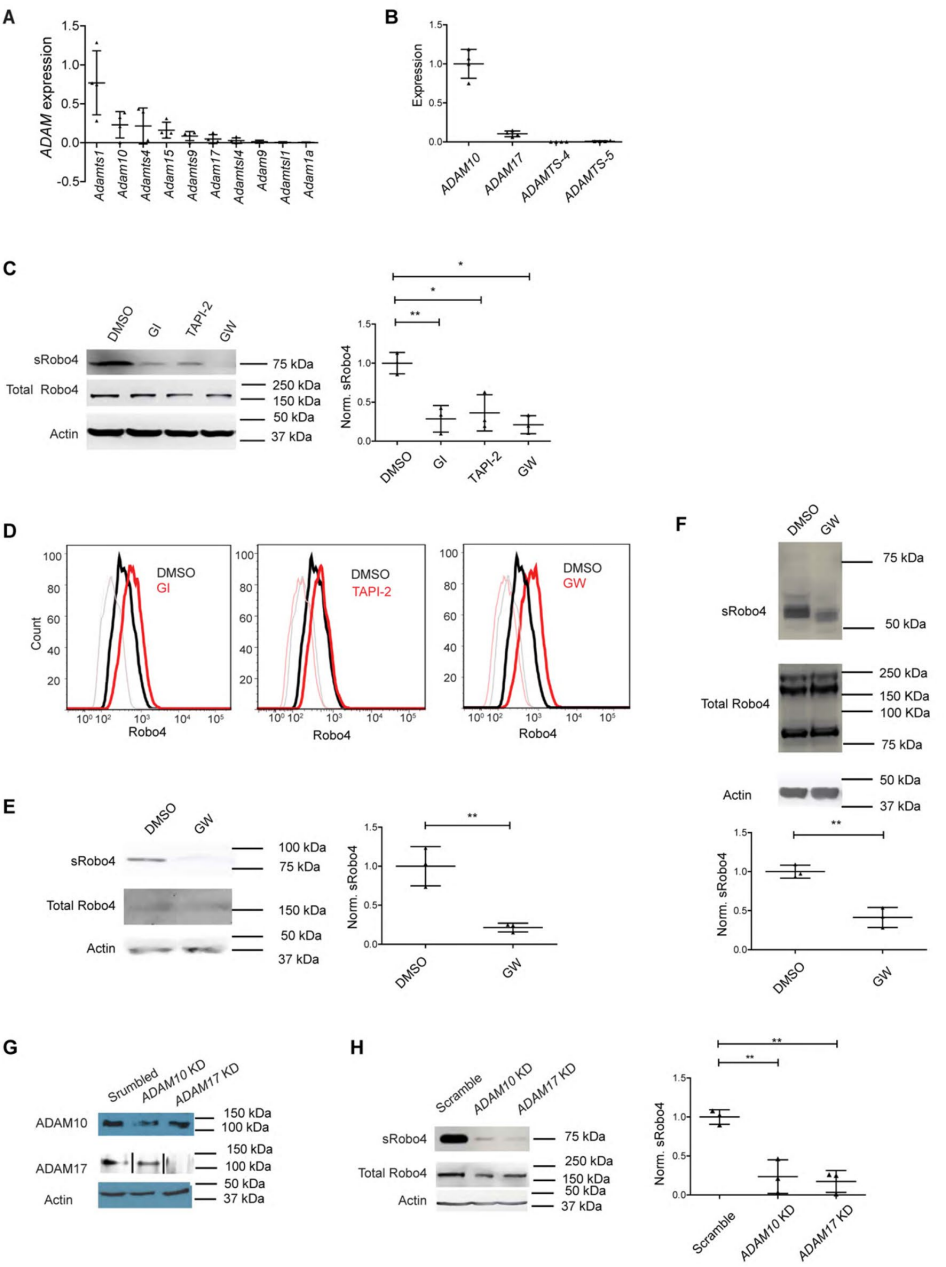
ADAM10 and ADAM17 are Robo4 sheddases. (**A**). Single-cell RNAseq transcriptome analysis of *ADAM* expression in dEC in adult C57BL/J mice^18^. *ADAM* expression was normalized to *GAPDH* in the same cell, and the mean value in each mouse was calculated. The top 10 expressing *ADAMs* in dEC are plotted. The full list of analyzed *ADAM*s is included in the Methods. (**B**) Quantitative RT-PCR analysis determined the mRNA expressions of *ADAM10*, *ADAM17*, *ADAMTS-4*, and *ADAMTS-5* in a mouse dEC line, and the data were normalized to *ADAM10* expression. (**C**–**F**) Pharmacological inhibition of ADAM10, ADAM17, or both blocked Robo4 shedding in dEC (**C**), mouse lung endothelial cells (**E**), and primary HUVECs (**F**) and led to corresponding increased cell surface Robo4 (**D**). The endothelial cells were treated with GI, TAPI-2, or GW at 6 μM or vehicle (DMSO) for 6 h, and sRobo4 in conditioned medium was assessed and normalized to full-length Robo4 in the cell lysate. The data was further normalized to the DMSO group for comparison. The dEC cell surface Robo4 was assessed by flow cytometry after staining with an anti-Robo4 ectodomain antibody. Anti-Robo4 IgG and naïve IgG staining are drawn in heavy-bright and thin-faint lines, respectively, with corresponding colors. (**G**) Knockdown (KD) of *ADAM10* and *ADAM17*. dECs were transiently transfected with scramble shRNA or shRNA against *ADAM10* or *ADAM17*, and ADAM10 and ADAM17 expression in the shRNA-treated cells were assessed by Western blot with corresponding specific antibodies. Black bars separate lanes that are nonadjacent in the same blot. (**H**) Knockdown of *ADAM10* or *ADAM17* each inhibited Robo4 shedding. sRobo4 in 6-h conditioned media was assessed by Western blot. The data represent 3 independent experiments and are presented as mean ± SD. The student’s t-test was performed for two-group comparisons. *p < 0.05; **p < 0.01.

The ADAM proteases ADAM10 and ADAM17 have been studied in ectodomain shedding of endothelial cell surface proteins, such as vascular endothelial cell growth factor receptor 2 (VEGFR2)^14,15^ and cadherin 5 (Cdh5)^16,17^. As of the Robo receptors, the Drosophila Robo is shed by *kuzbanian*, the homolog of mammalian ADAM10, and overexpression of a dominant-negative human ADAM10 blocked Slit2-induced plasma membrane recruitment of Sos protein and morphological change in Robo1-expressing HEK cells^18^. We hypothesized that ADAMs are responsible for Robo4 shedding. We previously determined Slit3-Robo4 signaling is required for angiogenesis in developing diaphragm^11^, and the constitutive Robo4 shedding occurs in our derived mouse dEC line (Fig. 1). Therefore, we applied dEC as the leading test model in the following studies. We first analyzed a publicly available single-cell RNAseq dataset which contains the gene expression profile of the mouse diaphragmatic endothelium^19^ and detected high *ADAM10* and *ADAM17* expression (Fig. 2A). We then performed quantitative RT-PCR analysis to examine mRNA expressions of *ADAM10*, *ADAM17*, and metalloproteinases *ADAMTS-4* and *ADAMTs-5* in the dEC line. *ADAM10* and *ADAM17* mRNAs were expressed in the dEC (Fig. 2B). To determine if ADAM10 and ADAM17 shed Robo4 from the endothelial cell surface, dEC were treated with GI254023x (GI), an ADAM10-specific inhibitor, TAPI-2, an inhibitor specific for ADAM17, or GW280264x (GW), a duo inhibitor for both ADAM10 and ADAM17. Accordingly, the three inhibitors each potently inhibited Robo4 shedding in dEC (Fig. 2C) and increased cell surface Robo4 (Fig. 2D). Similarly, the inhibition of ADAM10 and ADAM17 with GW also diminished Robo4 shedding from mouse lung endothelial cells (Fig. 2E) and primary human umbilical vein endothelial cells (HUVEC, Fig. 2F). To alternatively confirm these findings, *ADAM10* or *ADAM17* was transiently knocked down in dEC (Fig. 2G). Knockdown of *ADAM10* or *ADAM17* each attenuated Robo4 shedding (Fig. 2H). Taken together, these results show that ADAM10 and ADAM17 are Robo4 sheddases.

### Inhibition of ADAM10 and ADAM17 reduces Robo4 C-terminal fragment generation, and ADAM10 and ADAM17 co-localize with Robo4 in endothelial cells

**Figure 3:**
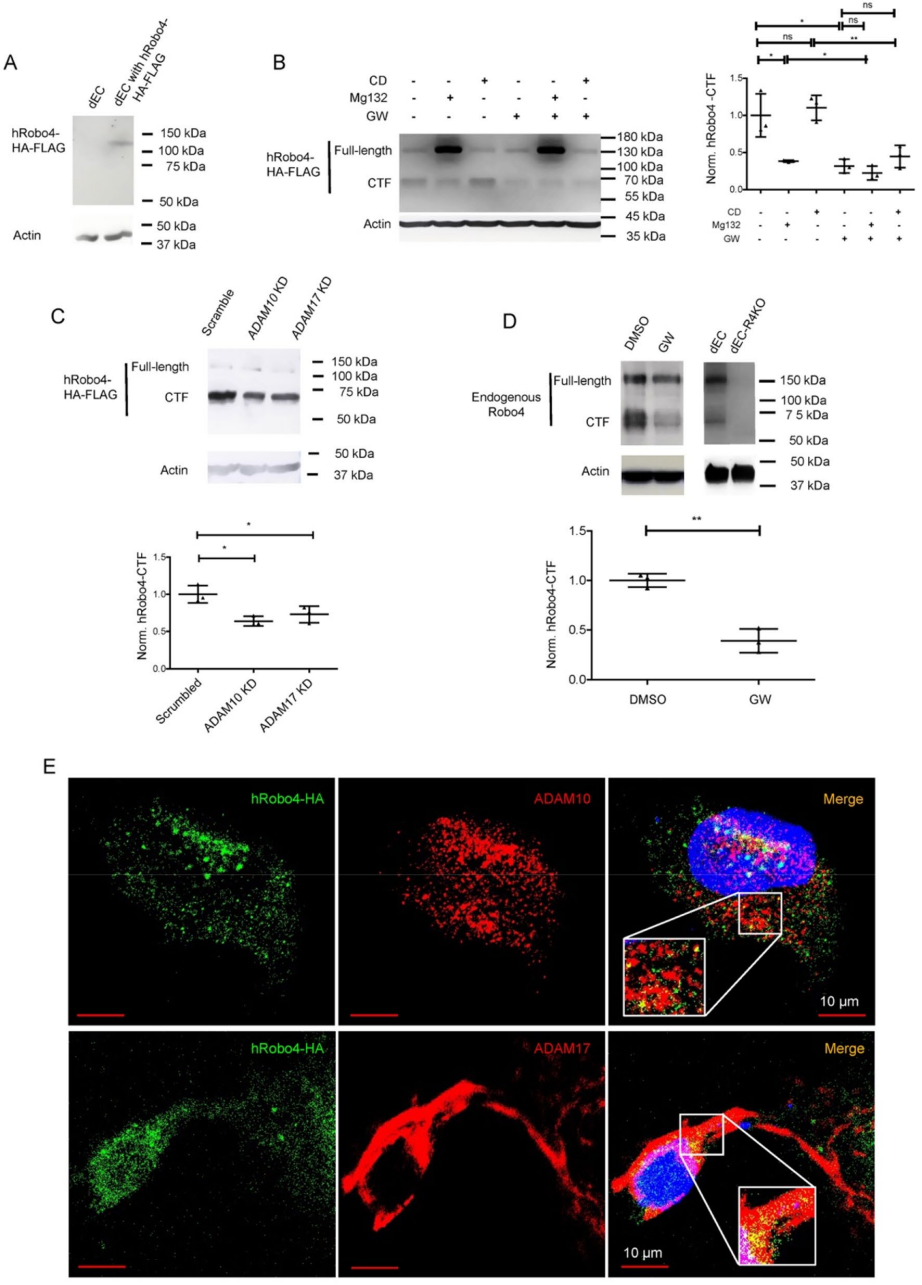
Inhibition of ADAM10 and ADAM17 increases Robo4 C-terminal Fragment, and Robo4 co-localizes with ADAM10 and ADAM17 in endothelial cells. (**A**) Human Robo4-HA-FLAG expression. The expression of hRobo4-HA-FLAG in dEC lysate was probed with an anti-human Robo4 ectodomain antibody in Western blot. A 130 KDa band was detected. (**B**) Pharmacological inhibition of ADAM10 and ADAM17 decreased Robo4-CTF. hRobo4-HA-FLAG transiently expressing dECs were treated with vehicle control DMSO or GW (6 μM) with or without Mg132 (12 μM) or chloroquine diphosphate (CD, 50 μM). The cell lysates were probed with an anti-FLAG antibody. The hRobo4 CTF was normalized to β-actin and further normalized to the control group. GW and Mg132 decreased hRobo4-CTF. (**C**) Knockdown of *ADAM10* or *ADAM17* decreased Robo4 CTF. The dECs were co-transected with hRobo4-HA-FLAG and scrambled or *ADAM* knockdown constructs and then probed with an anti-FLAG antibody in western blot. (**D**) Inhibition of ADAM10 and ADAM17 decreased endogenous Robo4-CTF. Vehicle DMSO or GW (6 μM)-treated dECs were lysed and probed with an anti-mouse Robo4 CTF antibody in western blot. No Robo4 bands were detected in the *Robo4* KO dEC cell lysate. (**E**) Robo4 co-localizes with ADAM10 and ADAM17. dECs were transiently expressed with hRobo4-HA-FLAG and stained for HA and endogenous ADAM10 or ADAM17 with corresponding antibodies. The merge yellow fluorescence shows Robo4-HA (green) co-localized with ADAM10 or ADAM17 (red). Robo4-HA-FLAG co-localized with ADAM10 or ADAM17 with overlap coefficients of 0.900 and 0.834, respectively. The data represent 3 independent experiments and are presented as mean ± SD. The student's t-test was performed for a two-group comparison. *p < 0.05; **p < 0.01.

Ectodomain shedding of cell surface transmembrane protein also generates a membrane-anchored cytosolic domain. Therefore, ectodomain shedding of transmembrane cell surface molecule can also be determined by examining the generation of its c-terminal fragment (CTF), as observed in the Robo1 and VEGFR2 shedding^20,21^. To assess Robo4-CTF generation, we transiently expressed human Robo4 with a C-terminal HA-FLAG duo tag (hRobo4-HA-FLAG) in the mouse dEC. The transient expression generated a 130 kDa hRobo4-HA-FLAG protein as detected by an anti-human Robo4 ectodomain antibody (Fig. 3A). To examine hRobo4-CTF, we probed with an anti-FLAG antibody. Except for the 130 kDa band, an additional protein band at 65 kDa was detected (Fig. 3B). We believed that the 130 kDa protein represented the full-length hRobo4-HA-FLAG and the 65 kDa protein was the Robo4-CTF. To confirm that ADAM10 and ADAM17 are the sheddases to produce the Robo4-CTF and determine the protein degradation pathway involved, transient hRobo4-HA-FLAG expressing dECs were treated with ADAM10- and ADAM17 duo inhibitor GW in the presence or absence of lysosome inhibitor chloroquine diphosphate or proteasome inhibitor Mg132 for 6 h. As expected, GW decreased hRobo4-HA-FLAG CTF (Fig. 3B). Mg132, but not chloroquine diphosphate, inhibited Robo4-CTF generation, showing that the Robo4-CTF degradation is mainly through the proteasome pathway. We also transiently expressed hRobo4-HA-FLAG in ADAM10- or ADAM17 knocked-down dECs. Knockdown of *ADAM10* or *ADAM17* each reduced hRobo4-HA-FLAG CTF (Fig. 3C). We similarly assessed the endogenous Robo4-CTF in dECs with an antibody specific for the CTF of mouse Robo4. Consistent with the human Robo4 study findings, GW reduced the endogenous mouse Robo4-CTF (Fig. 3D). These results confirmed that ADAM10 and ADAM17 are Robo4 sheddases and also revealed that the degradation of the generated Robo4-CTF is mainly through the proteasome pathway. Last, we stained transiently expressed hRobo4-HA-FLAG and endogenous ADAM10 and ADAM17 in dECs. Robo4-HA-FLAG co-localized with ADAM10 or ADAM17 with overlap coefficients of 0.900 and 0.834, respectively (Fig. 3E), supporting the model that ADAM10 and ADAM17 physically interact with Robo4 to execute as Robo4 sheddases.

### Robo4-Ig inhibits angiogenic Slit3-Robo4 signaling in vitro and in vivo

**Figure 4:**
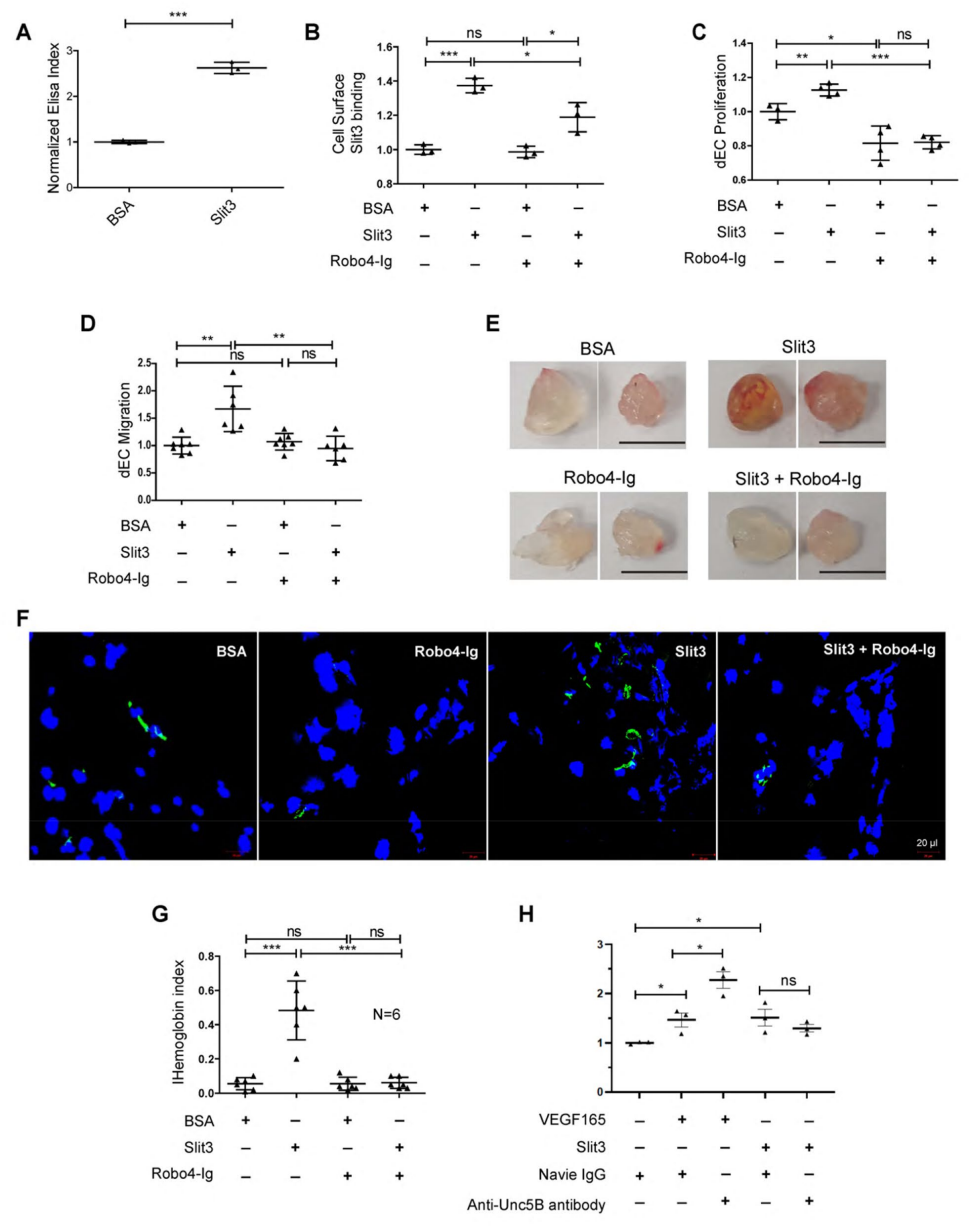
Robo4-Ig inhibits Slit3-induced angiogenesis. (**A**). Robo4-Ig bound Slit3. Robo4-Ig was immobilized in an ELISA plate and incubated with His-tagged Slit3. The bound Slit3 was measured by ELISA using an HRP-conjugated anti-His antibody. BSA was used as a background control and for normalization. (**B**). Robo4-Ig inhibited Slit3 binding to dEC surface. Confluent dECs were fixed and incubated with His-tagged Slit3 or BSA in the absence or presence of Robo4-Ig. The cell surface-bound Slit3 was quantified by ELISA using an HRP-conjugated anti-His antibody. BSA was used as a background control and for normalization. (**C**, **D**) Robo4-Ig inhibited Slit3-induced dEC proliferation and migration. Slit3-induced dEC proliferation in DMEM containing 0.2% serum (36-h, **C**) and Slit3-induced transwell cell migration in serum-free DMEM (9-h, **D**) were determined in the absence or presence of Robo4-Ig. BSA was included as a background control. (**E**–**G**) Robo4-Ig inhibited Slit3-induced neovascularization in matrigel plug assay. The Slit3- or BSA-supplemented growth factor reduced matrigel with or without Robo4-Ig was implanted *s.c.* into adult female mice, and the plugs were harvested two weeks later. Representative gross images (**E**) and neo-vasculature staining (**F**) are shown , including CD31 staining (green) of endothelial cells, DAPI staining (blue) of cellularity (**F**), and hemoglobin content in the Matrigel plugs (**G**), N = 6 per group. (**H**). Anti-Unc5B neutralizing antibody treatment in the VEGF165—or Slit3-induced dEC migration. VEGF165- (100 ng/ml) or Slit3-induced dEC transwell cell migration in serum-free DMEM was determined in the presence of native IgG or anti-Unc5B neutralizing IgG antibody (2 μg/ml). The data shown in A-D and H represent 3 independent experiments. All the data are presented as mean ± SD. The student's t-test was performed for a two-group comparison and ANOVA for multiple groups. ns, not significant; *p < 0.05; **p < 0.01; ***p < 0.001.

Within the Robo ectodomain, the two Ig domains mediate interaction with its ligand Slit2^22^, and the two fibronectin type III repeats bind to cell adhesion molecules^23^. Our MS analysis detected that sRobo4 contains both Ig1 and Ig2 domain sequences (Fig. 1B), suggesting that sRobo4 can potentially interact with Slit3 and modulate Slit3 activity. To study sRobo4’s effect on angiogenic Slit3-Robo4 signaling, we generated a recombinant Robo4 ectodomain (Robo4-Ig) which contains both the Ig1 and Ig2 domains as a substitute of the sRobo4 for the functional study. Slit3 showed strong binding to immobilized Robo4-Ig showing Ig1 and Ig2 is the Slit3-binding site within Robo4 (Fig. 4A). Robo4-Ig inhibited Slit3’s binding on the dEC cell surface (Fig. 4B) and potently impeded dEC’s constitutive and Slit3-induced proliferation (Fig. 4C). Robo4-Ig also inhibited Slit3-induced dEC migration (Fig. 4D). In vivo, Slit3 induced robust neovascularization in the matrigel plug angiogenesis assay as we reported^3,11^. This pro-angiogenic effect was abolished by Robo4-Ig, including diminishing reddish-looking, endothelial cells and hemoglobin content (Fig. 4E–G). Robo4 was reported to bind Unc5B, a vascular Netrin receptor, to maintain vessel integrity and inhibits VEGF-induced angiogenesis^6^. dEC also expresses Unc5B (Supplementary Fig. 2). We further tested if Unc5B affects Slit3-induced dEC functions in cell migration assay. Neutralizing Unc5B with an anti-Unc5B polyclonal antibody increased VEGF165-induced dEC migration but did not alter Slit3-induced dEC migration (Fig. 4H), indicating that the inhibitory effects of Robo4-Ig on Slit3-induced dEC proliferation and migration do not depend on the Unc5B pathway. Taken together, these in vitro and in vivo experimental results show sRobo4 competes with cell surface Robo4 for ligand Slit3 binding, thereby inhibiting Slit3-Robo4-mediated angiogenesis.

### Slit3 induces Robo4 internalization to attenuate Robo4 shedding

**Figure 5:**
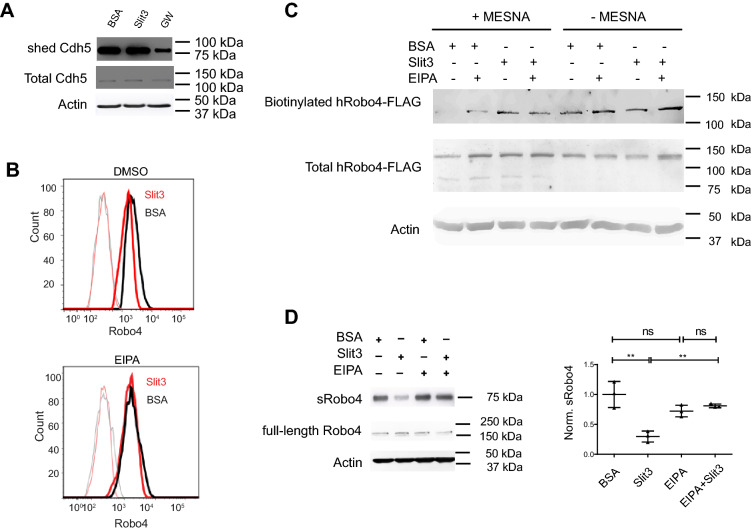
Slit3 down-regulates cell surface Robo4 and induces Robo4 internalization. (**A**) GW but not Slit3 reduced Cdh5 shedding. dECs in serum-free cultured were supplemented with BSA or Slit3 with or without GW, the duo-inhibitor for ADAM10 and ADAM. The conditioned media and cell lysate were probed with an anti-Cdh5 antibody in Western blot. (**B**) Slit3 reduced cell surface Robo4, and this was blocked by EIPA. Starved dECs were treated with Slit3 or BSA in the absence or presence of EIPA, and then cell surface Robo4 was assessed by flow cytometry after staining with an anti-Robo4 ectodomain antibody. Anti-Robo4 IgG and naïve IgG staining are drawn in heavy-bright and thin-faint lines, respectively, with corresponding colors. (**C**) Slit3 induced cell surface Robo4 internalization, and this was blocked by EIPA. After biotinylation of cell surface proteins at 4 °C, the hRobo4-HA-FLAG expressing dECs were treated with Slit3 or BSA in the absence or presence of EIPA at 37 °C. Cells were lysed immediately (total biotinylated protein preserved) or after MENSA treatment (MESNA cleaves uninternalized biotinylated protein), and biotinylated proteins were pulled down using streptavidin-coated beads. hRobo4-HA-FLAG were probed in western blot with anti-FLAG antibody. (**D**) Slit3 inhibited Robo4 shedding, and this was restored by EIPA. dECs were treated with Slit3 or BSA in the absence or presence of EIPA, and then sRobo4 in conditioned medium, and total Robo4 and actin in cell lysates were probed. sRobo4 was normalized to the total Robo4. The data represent 3 independent experiments and are presented as mean ± SD. The student's t-test was performed for a two-group comparison. ns, not significant; *p < 0.05; **p < 0.01.

Ectodomain shedding of cell surface receptors can be induced by its ligand via activation of ADAMs, coincident with enhanced shedding of other ADAM substrates, as exampled for VEGFR2’s shedding upon VEGF-A treatment^24^. Meanwhile, VEGF-A induces VEGFR2 internalization^25^, and the constitutive endocytosis shields VEGFR2 from the shedding^21^. To test if Slit3 affects Robo4 shedding, we first examined if Slit3 influences ADAMs’ activity by probing the ectodomain shedding of Cdh5, a known substrate for both ADAM10 and ADAM17 on the endothelial cell surface^16,17^. The Cdh5 shedding was inhibited by GW but not affected by Slit3 (Fig. 5A). Slit3 also did not alter the expression of the total ADAM10 and ADAM17 protein levels in Western blotting analysis (Supplemental Fig. 3A–C) or the abundance of ADAM10 and ADAM17 on dEC cell surface (Supplemental Fig. 3D,E). These data show that Slit3 does not alter ADAM10 and ADAM17 expression or activity to regulate Robo4 shedding. Second, we examined if Slit3 regulates cell surface Robo4 level and observed Slit3 reduced cell surface Robo4 (Fig. 5B). Interestingly, this cell surface Robo4 reduction was inhibited by co-incubation with 50 μM EPIA, a macropinocytosis inhibitor. Meanwhile, Slit3 enhanced the internalization of the biotinylated cell surface Robo4 into cells (Fig. 5C). These observations showed Slit3 reduced cell surface Robo4 by inducing Robo4 internalization. Since Slit3 did not alter the activity and expression of ADAM10 and ADAM17 but decreased the abundance of cell surface Robo4, we postulated Slit3 inhibits Robo4 shedding. Indeed, sRobo4 was reduced in the conditioned medium collected from Slit3-treated dEC, and this effect was inhibited by blocking endocytosis with EIPA (Fig. 5D). Taken together, these results show that Slit3 induces Robo4 internalization to shield cell surface Robo4 shedding from ADAM10 and ADAM17, thereby inhibiting Robo4 shedding.

## Discussion

**Figure 6:**
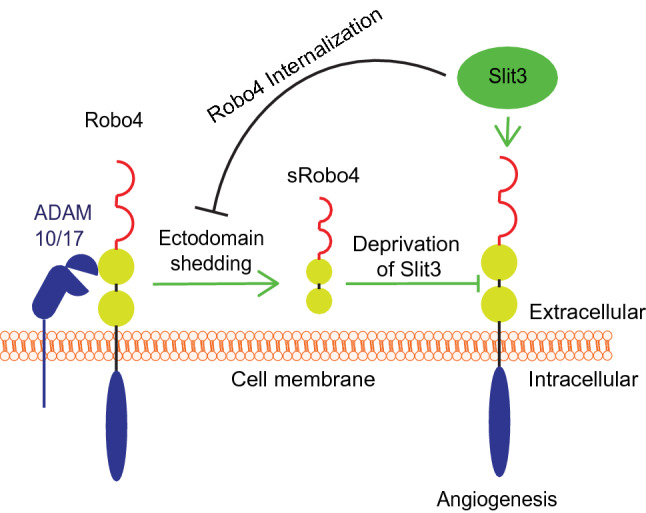
sRobo4 generation and its role in angiogenic Slit3-Robo4 signaling. Under the unstimulated condition, the Robo4 ectodomain is constitutively cleaved by ADAM10 and ADAM17 to generate sRobo4. The generated sRobo4 blocks Slit3-Robo4 interaction, thereby inhibiting angiogenic Slit3 signaling. Meanwhile, Slit3 inhibits Robo4 shedding by inducing Robo4 internalization to shield the receptor from shedding.

Robo4 plays an essential role in angiogenesis and inflammation, but how Robo4 signaling is regulated remains incompletely understood. This study delineated that ectodomain shedding represents a novel regulatory mechanism of Robo4 signaling (Fig. 6). Cell surface Robo4 undergoes constitutive proteolytic cleavages by ADAM10 and ADAM17 to generate sRobo4. As a result, sRobo4 can inhibit angiogenic Slit3-Robo4 signaling, manifesting a negative regulation of Robo4 signaling. Meanwhile, Slit3 enhances Robo4 endocytosis, thereby shielding Robo4 from shedding, showing Slit3 inhibits Robo4 shedding to enhance Robo4 signaling. These findings advanced our understanding of Robo4 signaling and its regulation.

Cell surface protein shedding can occur constitutively or be induced by their ligands or chemical agents such as phorbol 12-myristate 13-acetate (PMA). We observed Robo4 sheds constitutively. Intriguingly, the ligand Slit3 does not induce, instead inhibits Robo4 shedding via a mechanism of enhancement of cell surface Robo4 internalization, revealing a novel pathway to regulate Robo4 shedding. We also tested two common shedding activators, including PMA and lipopolysaccharide (LPS). Both PMA and LPS induced syndecan-1 shedding as reported in the literature^26^, but not Robo4 in dECs (Supplemental Fig. 4). Clinical studies have reported that soluble Robo4 in patient plasma raised significantly 2 h after cardiac surgery^8^ and might be one of the plasma biomarkers in the clinical course of cerebral cavernous malformations^9^. Our subsequent study would determine if upregulated ADAM10 and/ADAM17 expression and activity are the underlying mechanism for the raised Robo4 in circulation in these pathological conditions and the possible pathogenic roles of sRobo4 in the diseases.

By analyzing single-cell RNAseq and experimental examination with endothelial cells from mouse diaphragm and lung, and primary HUVECs, we identified ADAM10 and ADAM17 are the Robo4 sheddases in endothelial cells. Our single-cell RNAseq analysis also observed MMP2 expression in the diaphragm endothelium (data not shown). We have not tested if MMP2 sheds Robo4 from endothelial cells. Considering the high efficacy of ADAM10/17 duo inhibitor GW to block Robo4 shedding, we postulate that ADAM10 and ADAM17 are the major Robo4 sheddases, at least for constitutive Robo4 shedding under an unchallenged condition. As mentioned above, it would be exciting to investigate if the expressions of ADAM10, ADAM17, MMP2, and other MMPs are altered in endothelial cells in pathological conditions, such as cardiac surgery and cavernous cerebral malformations, thereby influencing Robo4 shedding to affect Robo4 signaling, attributing to the related pathogenesis.

Ectodomain shedding is a common mechanism to regulate ligand-cell surface receptor signaling implicated in axon guidance and angiogenesis. For example, cleavage of the Netrin receptor Deleted in Colorectal Cancer attenuates Netrin signaling^27^, and the shedding of VEGFR2 and lymphatic vessel endothelial hyaluronan receptor I regulate VEGF signaling in angiogenesis and lymphangiogenesis, respectively^21,24,28^. In the present study, we observed Robo4 sheds from endothelial cells, and sRobo4 inhibits Slit3’s binding on the endothelial cell surface and, in consequence, blocks the Slit3-Robo4 signaling-mediated angiogenesis, showing Robo4 shedding negatively regulates angiogenic Slit3-Robo4 signaling. Meanwhile, our studies also determined that Slit3 enhances Robo4 endocytosis, thereby shielding Robo4 from shedding, manifesting a pathway by which Slit3 inhibits Robo4 shedding to enhance Robo4 signaling. Slit3 is not the only ligand for Robo4. Slit2 binds Robo4, and Slit2-Robo4 signaling promotes or inhibits angiogenesis depending on cell type and tissue context^4–6,10^. Annexin A2 also binds Robo4 and modulates Robo4's interaction with Paxillin and downstream ARF6 activation^29^. In further studies, we will determine if sRobo4 blocks Slit2-Robo4 and Annexin A2-Robo4 signaling and if these ligands regulate Robo4 shedding.

Robo4 acts as a ligand of Unc5B, and the Robo4-Unc5B signaling blocks VEGF-induced vascular permeability to maintain vessel integrity^6^. In our study, we tested if Unc5B involves in the inhibitory effect of Robo4-Ig on Slit3-induced endothelial cell migration. We observed that neutralizing Unc5B with a functional blocking antibody enhanced VEGF-induced endothelial cell migration but did not affect Slit3-induced endothelial cell migration. These results indicate that the inhibitory effect of Robo4-Ig on Slit3-mediated endothelial angiogenesis does not depend on the Unc5B pathway. In the reported Robo4-UNC5B signaling model, the ligand-receptor interaction occurs between neighbor endothelial cells^3^. The occurrence of Robo4 ectodomain shedding, discovered in current study, suggests Robo4 may also activate Unc5B signaling through autocrine and paracrine manners in the vasculature.

Within the Robo ectodomain, the two Ig domains mediate its interaction with ligand Slit2^22^, and the two fibronectin type III repeats bind to cell adhesion molecules^23^. Our proteomics analysis determined that the sRobo4 contains both the Ig and the fibronectin domains. To be more clearly determine how sRobo4 affects Slit3-Robo4 signaling, our study applied a recombinant Robo4-Ig which contains only the Ig1 and Ig2 domains without the fibronectin domains. We observed that Slit3 bound firmly to immobilized Robo4-Ig, showing directly that the Ig domains of Robo4 are the bindings site of Slit3, and Robo4-Ig potently inhibits Slit3-induced angiogenesis in vitro and in vivo. Since the sRobo4 also contains the fibronectin domains. There is a possibility that sRobo4 may also interfere with the fibronectin domain mediated-cell adhesion, which needs to be addressed in future studies.

We observed endogenous full-length Robo4 expressed as a 160 kDa protein, whereas transiently expressed full-length hRobo4-HA-FLAG expressed as a 130 kDa protein. We postulate that the size difference is because of insufficient post-translational modification, such as glycosylation, as hRobo4-HA-FLAG was transiently over-expressed. Consistently, western blotting probing C-terminal of endogenous Robo4 detected the full-length mouse Robo4 as two distinct bands with one minor band at 130 kDa (Fig. 3D). In contrast, full-length hRobo4-HA-FLAG was probed mainly as one 130 kDa band in the over-expressing cells (Fig. 3B). In addition, the sizes of sRobo4 from HUVECs are 55- and 60 kDa (Fig. 2F), distinct from what we observed in the mouse cell lines (Fig. 1A), and was close to the size of previously reported HUVEC-sRobo4^7^, suggesting the variation of sRobo4 size in mouse and human endothelial cells might also be because of species-specific post-translational modification or different levels of turnover.

In summary, our study showed that Robo4 shedding occurred constitutively, and the identified ADAM10 and ADAM17 are Robo4 sheddases. Functional studies determined sRobo4 inhibits Slit3-Robo4 signaling in angiogenesis, and Robo4 shedding is hampered by its ligand Slit3. Our studies illuminated that Robo4 ectodomain shedding is a novel regulatory mechanism of Robo4 signaling and might be associated with human diseases.

## Experimental procedures

### Reagents

#### Plasmids, proteins, and chemicals

Human Robo4 cDNA (NM_019055.5) was cloned into NheI/NotI sites of pCDH-EF1-FHC plasmid (Addgene, #64874) to generate hRobo4-HA-FLAG construct. Plasmids psPAX2, (#12260) and pMD2.g (#12259) for lentiviral transfection were purchased from Addgene. ShRNA plasmids MSH028611-LVRU6GP, CSHCTR001-LVRU6GP, MSH026499-LVRU6GP were from Genecopeia. His-tagged recombinant mouse Slit3 (3629-SL-050) was from R&D Systems, PMA (ab120297) from Abcam, TAPI-2 (187034-31-7) from Cayman Chemical, GW282026X (AOB3632) from AOBIOUS Inc., Polybrene (sc-134220) from Santa Cruz Biotechnology, and GI254023x (260264-93-5), LPS (#L7770) and PEI (#764965) were from Sigma-Aldrich.

#### Antibodies

Anti-N-terminal mouse Robo4 antibody (Abcam, ab10547), anti-N-terminal human Robo4 antibody (R&D Systems, MAB2454), anti-FLAG antibody (Thermo Fisher Scientific, 14-6681-82), anti-HA antibody (Chromotek, #7c9-100), anti-intracellular Robo4 domain antibody (Santa Cruz Biotechnology, sc46497), anti-ADAM10-pro antibody (Abcam, ab39178), anti-ADAM10 antibody (Bioss, bs-3574R; LSbio, C497146-200; Novus, #NBP1-76973), anti-ADAM17 antibody (Bioss, 4236R; Novus #NBP2-61719), anti-Synecan-1 antibody (Santa Cruz Biotechnology, sc-5632), anti-actin antibody (Sigma Aldrich, A2228), anti-GAPDH antibody (R&D Systems, AF5718), polyclonal anti-Unc5B antibody (R&D Systems, AF1006), anti-N-terminal CDH5 (Thermo Fisher Scientific, 14-1441-82), anti-CD31 (BD, #550274), HRP-conjugated anti-His antibody (Alpha Diagnostic International, HISP12-HRP), HRP-conjugated goat anti-rabbit IgG antibody (Santa Cruz Biotechnology, sc-2030), HRP-conjugated donkey anti-goat IgG antibody (Santa Cruz Biotechnology, sc-2020), and HRP-conjugated goat anti-mouse IgG antibody (Invitrogen, 62-6520).

#### Other materials

Growth factor-reduced Matrigel (Corning, 356231). Thirty kDa protein filters (VWR, 89131-984 or Millipore UFC803024), ten kDa protein filters (VWR, 89132-008), CIM-plate 16 (ACEA, 05665817001), E-plate 16 (ACEA, 5469813001), iscript cDNA synthesis kit (Bio-Rad, 1708890), SYBR green kit (Bio-Rad, 1725270), Drabkin’s reagent (Sigma Aldrich, D5941-6VL), lenti-X concentrator (TaKaRa bio USA Inc., #631232), polybrene (Santa Cruz Biotechnology, #134220). The protein ladder was obtained from Bio-Rad (#161-0374) and TMB substrate from Thermo Fisher scientific (#N301). Propidium iodide (PI) was from Calbiochem (#537060).

### Endothelial cells

The mouse lung endothelial cell line and the mouse dEC line were generated by immortalizing primary cells isolated from adult C57/BL6 mice using SV40 large T-antigen^11,30–33^. The endothelial cell lines were cultured in DMEM with 10% FBS, penicillin–Streptomycin (100 µ/ml and 100 μg/ml, respectively) in a humidified cell culture incubator set at 5% CO_2_ and 37 °C. The primary HUVECs from ATCC (PCS-100-013) were cultured with the Endothelial Cell Growth Kit-BBE (ATCC® PCS-100-040) following the kit`s instruction.

### Preparation of concentrated conditioned media

To determine Robo4 shedding, 3 × 10^6^ endothelial cells were seeded onto a 150 mm cell culture plate and cultured for 12 h and then starved in 10 ml DMEM (hyclone, #sh30243.01) on an orbital rotator for 6 h. Following, the conditioned media was collected, centrifuged at 400 g for 5 min to remove cell debris, filtered through a 0.2 μm membrane filter, and concentrated to ~ 50 µl using a 30 kDa cut-off spin column. To test Slit3’s role in Robo4 shedding, dECs were starved for 6 h in serum-free DMEM, and then the media was replaced with fresh DMEM supplemented with BSA, Slit3 (1 μg/ml) or chemicals. The conditioned medium of 6-h culture was collected for shed Robo4 analysis.

### Western blotting

The total concentrated conditioned media (shed Robo4) from one 150 mm culture dish, or 2% of the corresponding cell lysate were separated in 7.5% or 12% SDS-PAGE, and then transferred to polyvinylidene difluoride membrane. After blocking, the membrane was probed with a primary antibody at 1 µg/ml followed by an HRP-conjugated secondary antibody at 300 ng/ml. Probed protein was detected via chemiluminescence using the Kwik quant imager system (Kindle Biosciences, D1001), and pixels of the protein band was quantified using ImageJ.

### Mass spectrometry (MS)

Shedding media was collected from 20 150 mm-dishes with confluent dECs. The media was first centrifuged at 400×*g* for 5 min and then filtered through a 100 kDa cut-off protein filter (Millipore, #C7715) at 400×*g*. The flow-through was concentrated to 100 µl via spinning over spin columns with a 30 kDa cut-off. The concentrated conditioned medium was resolved in a 7.5% PAGE gel, and the 75 kDa region was excised. The gel band was reduced with dithiothreitol (DTT), alkylated with iodoacetamide (IAA), and digested with Trypsin/Lys-C overnight at 37 °C. Following digestion, peptides were extracted from the gel using 50% water/50% acetonitrile/0.1% formic acid. Peptide samples were entirely dried in a speed vac centrifuge and resuspended in 1% acetonitrile/0.1% formic acid. For LC–MS/MS, peptides were separated using a 50 cm C18 reversed-phase UHPLC column (Thermo Fisher Scientific) on an Ultimate3000 UHPLC (Thermo Fisher Scientific) with a 120-min gradient (2–32% acetonitrile/0.1% formic acid) and analyzed on a hybrid quadrupole-Orbitrap mass spectrometer (Q Exactive Plus, Thermo Fisher Scientific) using data-dependent acquisition (DDA) in which the top 10 most abundant ions were selected for MS/MS analysis. Full MS scans were performed with a mass range of 375–1500 m/z and a resolution of 70,000. MS/MS scans were acquired with a resolution of 17,500. Raw data files were processed in MaxQuant (www.maxquant.org) and searched against 55,471 proteins from the Uniprot Mouse Reference protein sequence database (Proteome ID: UP000000589, version May 4th, 2020). Search parameters included constant modification of cysteine by carbamidomethylation and the variable methionine oxidation and protein N terminus acetyl groups. The first search peptide tolerance for precursor ions was at 20 ppm, while the main search peptide tolerance was at 4.5 ppm. Mass tolerance for fragment ions was at 10 ppm. Proteins were identified using the filtering criteria of 1% protein and peptide false discovery rate. The protein content was analyzed using a Q Exactive™ Plus Hybrid Quadrupole-Orbitrap™ Mass Spectrometer in the Proteomics Core at the University of South Florida.

### Bioinformatics analysis

Single-cell RNAseq expressional profiles of diaphragmatic endothelial cells were extracted from a published dataset^19^. Cells containing 0 counts of *the GAPDH* gene were removed. The counts of ADAM genes were normalized to that of the *GAPDH* gene. The mean of the normalized counts was calculated for each mouse. Genes of the top 10 means were plotted (Fig. 2A). The list of the ADAM genes are: *Adam10*, *Adam11*, *Adam12*, *Adam15*, *Adam17*, *Adam18*, *Adam19*, *Adam1a*, *Adam1b*, *Adam2*, *Adam21*, *Adam22*, *Adam23*, *Adam24*, *Adam25*, *Adam26a*, *Adam26b*, *Adam28*, *Adam29*, *Adam3*, *Adam30*, *Adam32*, *Adam33*, *Adam34*, *Adam39*, *Adam4*, *Adam5*, *Adam6a*, *Adam6b*, *Adam7*, *Adam8*, *Adam9*, *Adamdec1*, *Adamts1*, *Adamts10*, *Adamts12*, *Adamts13*, *Adamts14*, *Adamts15*, *Adamts16*, *Adamts17*, *Adamts18*, *Adamts19*, *Adamts2*, *Adamts20*, *Adamts3*, *Adamts4*, *Adamts5*, *Adamts6*, *Adamts7*, *Adamts8*, *Adamts9*, *Adamtsl1*, *Adamtsl2*, *Adamtsl3*, *Adamtsl4*, *Adamtsl5*.

### Quantitative RT-PCR (qRT-PCR)

RNA isolated from dECs was reverse-transcribed with an iScript cDNA synthesis kit (Bio-Rad #1708890), followed by real-time PCR with SYBR Green kit (Biorad #1725270). Primers used are: ADAM10-f tcatgggtctgtcattgatgga; ADAM10-r tcaaaaacggagtgatctgcac; ADAM17-f aggacgtaattgagcgattttgg; ADAM17-r tgttatctgccagaaacttccc; ADAMTS4f. atgtgggcacagtgtgtgat; ADAMTS4r caaggtgagtgcttcgtctg; ADAMTS5f. ggcatcattcatgtgacacc; ADAMTS5r cgagtactcaggcccaaatg; GAPDH-r ccagtgagcttcccgttca; GAPDH-f gaacatcatccctgcatcca.

### ADAM10 and ADAM17 knockdown

shRNA plasmids from Genecopiea targeting ADAM10 (MSH028611-2-LVRU6GP), ADAM17 (MSH026499-3-LVRU6GP) or scrambled control (CSHCTR001-LVRU6GP) were transected into dEC via lentivirus. The ratio of the shRNA-expressing cell was evaluated by fluorescence. Batches with over 90% of fluorescent cells were used for downstream analysis.

### Flow cytometry

dECs were washed 3 times with pre-cooled DPBS and incubated with DPBS containing 1% BSA and 2 mM EDTA (DBE buffer) on ice for 10 min. Then cells were dissociated from the plates via pipetting. The dissociated cells were resuspended at 1.2 × 10^6^/ml in DBE buffer and incubated with primary antibody at 10 μg/ml on ice for 1 h. After washing, the cells were further incubated with a primary antibody followed by a secondary antibody at 5 µg/ml for 1 h. The cells were additionally stained with PI (1:500 dilution, CalBiochem #537060) for 20 min and then analyzed by flow cytometry using BD LSRII. The collected data were processed with Flowjo.

### Lentiviral expression of human Robo4 in dECs

Two million HEK293T cells were seeded onto a 10 cm plate and grew for 1 day in DMEM supplemented with 10% FBS. Then the HEK293T cells were transfected with a mixture of 3 transfection plasmids, including lentiviral plasmid carrying hRobo4-HA-FLAG, psPAX2 (addgene, #12260), and pMD2.g (addgene, #12260). Sixty microliter of PEI at 1 mg/ml was also added. After two day's culture, the viruses were harvested using Lenti-X concentrator following the recommended protocols. The hRobo4-HA-FLAG virus was added to a 6-well plate seeded with dECs. The dECs were allowed to grow for a week before upcoming experiments.

### Immunofluorescence to detect Robo4, ADAM, and CD31 expression

To determine Robo4 and ADAM10 or ADAM17 colocalization, the transiently hRobo4-HA-FLAG expressing dECs were washed with PBS and fixed with methanol (pre-cooled to − 20 °C) on ice. Following, the cells were incubated with a blocking solution containing 1 × DPBS, 0.5% Triton X-100, and 5% normal goat serum at room temperature for 1 h and then with rat-anti-HA (Chromotek, 7c9-100) and rabbit anti-ADAM17 (Novus Biologicals, NBP2-67179) or rabbit anti-ADAM10 (Novus, #NBP1-76973) antibody at 4 °C overnight. After washing with PBS, the cells were incubated for 1 h with Alexa Fluor 488- or Alexa Fluor 594-conjugated secondary IgG antibodies. Slides were then washed with PBS and mounted with fluorochrome mounting solution. To assess neovascularization in the Matrigel plug, the collected Matrigel plugs were fixed in 2-methyl butane pre-cooled in liquid nitrogen. 10 µm sections were blocked with a solution containing 1 × DPBS, 0.5% Triton X-100, and 5% normal goat serum at room temperature for 1 h and then incubated with rat anti-mouse CD31 antibody at 4 °C overnight, followed by Alexa Fluor 488-conjugated anti-rat secondary antibody for 1 h at room temperature. The images of the cultured cells and matrigel plug sections were captured with the Olympus FV10i confocal microscope (Tokyo, Japan). ImageJ JACop (Just Another Cocolization Plugin) was applied to determine the co-localization of Robo4 with ADAM10 and ADAM17 with a threshold = 50 for both green (Robo4) and red (ADAM10 or ADAM17) channels.

### Production of Robo4-Ig

The recombinant extracellular domain of human Robo4 (Robo4-Ig), including Ig 1 and 2 domains, was expressed as we expressed human Robo1 (46). In brief, human *Robo4 Ig1-2* cDNA was cloned into pGEn2 vector and transiently expressed in HEK293 cells^34^. Robo4-Ig-GFP-His in conditioned media was purified using a Ni–NTA superflow column (Qiagen, Valencia, CA). Fractions containing fluorescence were pooled and concentrated to ∼ 1 mg/ml using an ultrafiltration pressure cell membrane (Millipore, Billerica, MA) with a 10 kDa molecular weight cut-off. Following, the GFP-His tag was cleaved from Robo4-Ig-GFP-His protein by TEV protease digestion. The GFP-His tag and Tev-protease were removed through the second Ni–NTA column. Flow-through from the second run was collected and concentrated to 2 mg/ml before loading onto Superdex 75 for further purification. Fractions of purified Robo4-Ig were pooled and concentrated to 2 mg/ml and stored at − 80 °C.

### Binding of Slit3 to Robo4-Ig measured by ELISA^35–37^

The 96-well ELISA platelet was coated with 100 µl/well of Robo4-Ig at 0.2 μg/ml. After blocking with BSA (1% in PBS) overnight, the wells were incubated with 0.5 μg/ml BSA or His-tagged Slit3 for 1 h. After three times washing with PBS, the wells were incubated with HRP-conjugated anti-His antibody at 1:3000 dilution in PBS for 1 h at room temperature. After washing three times with PBS, the wells were then incubated with TMB substrate, and A450nm was measured after inactivating HRP activity with 50 μl/well 2 M sulfuric acid.

### Slit3 endothelial cell surface protein binding

1 × 10^4^ dECs per well were seeded in 96-well plates 12 h before the assay, then the cells were fixed with PFA 4% for 20 min, RT, and washed gently with PBS three times, 5 min each. Following, the cells were blocked in PBS with 0.5% BSA (PH 7.4) (PBS-BSA) for 30 min, incubated with 1 μg/ml BSA, or Slit3 w/o 20 μg/ml Robo4-Ig in PBS-BSA for 2 h. After that, the cells were washed with PBS-BSA 3 times, 5 min each, and incubated with 1:3000 diluted anti-His-HRP in PBS-BSA for 1. After washing with PBS-BSA three times, 5 min each, the wells were incubated with TMB substrate, and A450nm was measured after inactivating HRP activity with 50 μl/well 2 M sulfuric acid.

### Endothelial cell migration and proliferation assays

For cell migration assay, starved dECs were loaded onto each well (20,000 dECs) with Slit3 (1 μg/ml), BSA (1 μg/ml) or VEGF165 (100 ng/ml) in the absence or presence of Robo4-Ig at 20 μg/ml, naïve IgG or polyclonal anti-Unc5B IgG antibody (2 μg/ml) in serum-free DMEM in CIM-16 plates. The index of cell migration was collected at ~ 9 h. For cell proliferation assay, dECs were loaded onto each well (5,000 dECs) with Slit3 or BSA at 1 μg/ml in the absence or presence of Robo4-Ig at 20 μg/ml in DMEM containing 0.2% FBS in E-16 plates, and 36-h cell proliferation was measured. The cell migration or proliferation was measured with the RTCA-DP instrument (ACEA biosciences).

### Matrigel plug angiogenesis assay

Mice on a C57BL/6 background (6–8 weeks old, female) were obtained from the Jackson Laboratory. The in vivo Matrigel plug angiogenesis assay experimental procedure was approved by the University of South Florida Institutional Animal Care and Use Committee following the Association for Assessment and Accreditation of Laboratory Animal Care and ARRIVE guidelines. In brief, pre-cooled 0.5 ml matrigel mixed with Slit3 or BSA (1 µg) without or with Robo4-Ig (10 µg) was injected s.c. into both the left and right sides of the caudal ventral area of mice. The Matrigel plugs were harvested two weeks post the gel injection, weighed, photographed, and homogenized. The solution was spun at 10,000×*g* for 15 min. The hemoglobin content in the supernatant was measured using Drabkin’s reagent following the kit`s instruction (Sigma D5941-6VL).

### Cell surface Robo4 internalization

Transiently transected hRobo4-HA-FLAG dECs at 90% confluent were starved in DMEM for 7 h. Then their cell surface proteins were biotinylated with Sulfo-NHS-SS-biotin (0.25 mg/ml in DPBS, pH 7.4) at 4 °C for one hour following the suggested protocol (Thermo Fisher Scientific, #21331). The cells were further treated with Slit3 or BSA at 1 μg/ml in the presence of vehicle control DMSO or EIPA (25 μM) at room temperature for 10 min, and then for another 30 min r at 37 °C with 5% CO_2_. Following, the cells were lysed with RIPA buffer (no MESNA) or incubated with ice-cold 100 mM MESNA (Thermo Fisher Scientific, #50-163-8016) at 100 mM in DPBS, pH7.4 twice, 15 min each time to remove cell surface biotin. After four additional washes with ice-cold DPBS, cells were lysed with RIPA buffer (MESNA group). Biotinylated proteins were pulled down from cell lysates (both “No MESNA” and MENSA-treated samples) using Neutravidin agarose (Thermo Fisher Scientific, #29201) at 4 °C overnight and then probed with an anti-FLAG antibody in western blot. The total cell lysates were similarly analyzed.

### Quantification and statistical analysis

The western blots were quantified using ImageJ. Statistical analysis was carried out with Prism 8 for Macintosh. All data were shown as mean ± SD and analyzed through a student’s t-test between two groups or ANOVA for multiple groups. In all tests, P-value < 0.05 was statistically significant.

## Supplementary Information

Supplementary Information.

## Data Availability

All the data are contained within the article.

## Abbreviations

ADAM: A disintegrin and metalloproteinase
MBEC: Mouse brain endothelial cell
CD: Chloroquine diphosphate
CTF: C-terminal fragment
dEC: Diaphragm endothelial cell
GI: GI254023x
GW: GW280264x
MS: Mass spectrometry
PMA: Phorbol 12-myristate 13-acetate
Robo4-Ig: Recombinant Robo4 containing only Ig1 and Ig2 domains
sRobo4: Shed Robo4
VEGF: Vascular endothelial growth factor
VEGFR2: Vascular endothelial growth factor receptor 2
LPS: Lipopolysaccharide
FL-Robo4: Full-length Robo4
dEC-R4KO: Diaphragm endothelial cells with Robo4 knockout
hRobo4-HA-FLAG: Human Robo4 with c-terminal HA and FLAG tag

## References

[1] Park KW, Morrison CM, Sorensen LK, Jones CA, Rao Y, Chien CB (2003). Robo4 is a vascular-specific receptor that inhibits endothelial migration. Dev. Biol..

[2] Bedell VM, Yeo SY, Park KW, Chung J, Seth P, Shivalingappa V (2005). roundabout4 is essential for angiogenesis in vivo. Proc. Natl. Acad. Sci. USA..

[3] Zhang B, Dietrich UM, Geng JG, Bicknell R, Esko JD, Wang L (2009). Repulsive axon guidance molecule Slit3 is a novel angiogenic factor. Blood.

[4] Marlow R, Binnewies M, Sorensen LK, Monica SD, Strickland P, Forsberg EC (2010). Vascular Robo4 restricts proangiogenic VEGF signaling in breast. Proc. Natl. Acad. Sci. USA..

[5] Jones CA, London NR, Chen H, Park KW, Sauvaget D, Stockton RA (2008). Robo4 stabilizes the vascular network by inhibiting pathologic angiogenesis and endothelial hyperpermeability. Nat. Med..

[6] Koch AW, Mathivet T, Larrivee B, Tong RK, Kowalski J, Pibouin-Fragner L (2011). Robo4 maintains vessel integrity and inhibits angiogenesis by interacting with UNC5B. Dev. Cell..

[7] Zhang F, Prahst C, Mathivet T, Pibouin-Fragner L, Zhang J, Genet G (2016). The Robo4 cytoplasmic domain is dispensable for vascular permeability and neovascularization. Nat. Commun..

[8] Burke-Gaffney A, Svermova T, Mumby S, Finney SJ, Evans TW (2014). Raised plasma Robo4 and cardiac surgery-associated acute kidney injury. PLoS ONE.

[9] Girard R, Zeineddine HA, Koskimaki J, Fam MD, Cao Y, Shi C (2018). Plasma biomarkers of inflammation and angiogenesis predict cerebral cavernous malformation symptomatic hemorrhage or lesional growth. Circ. Res..

[10] Suchting S, Heal P, Tahtis K, Stewart LM, Bicknell R (2005). Soluble Robo4 receptor inhibits in vivo angiogenesis and endothelial cell migration. FASEB J..

[11] Zhang B, Xiao W, Qiu H, Zhang F, Moniz HA, Jaworski A (2014). Heparan sulfate deficiency disrupts developmental angiogenesis and causes congenital diaphragmatic hernia. J. Clin. Invest..

[12] Weber S, Saftig P (2012). Ectodomain shedding and ADAMs in development. Development.

[13] Dreymueller D, Pruessmeyer J, Groth E, Ludwig A (2012). The role of ADAM-mediated shedding in vascular biology. Eur. J. Cell. Biol..

[14] Jin Y, Liu Y, Lin Q, Li J, Druso JE, Antonyak MA (2013). Deletion of Cdc42 enhances ADAM17-mediated vascular endothelial growth factor receptor 2 shedding and impairs vascular endothelial cell survival and vasculogenesis. Mol. Cell. Biol..

[15] Donners MM, Wolfs IM, Olieslagers S, Mohammadi-Motahhari Z, Tchaikovski V, Heeneman S (2010). A disintegrin and metalloprotease 10 is a novel mediator of vascular endothelial growth factor-induced endothelial cell function in angiogenesis and is associated with atherosclerosis. Arterioscler. Thromb. Vasc. Biol..

[16] Schulz B, Pruessmeyer J, Maretzky T, Ludwig A, Blobel CP, Saftig P (2008). ADAM10 regulates endothelial permeability and T-Cell transmigration by proteolysis of vascular endothelial cadherin. Circ. Res..

[17] Weskamp G, Mendelson K, Swendeman S, Le Gall S, Ma Y, Lyman S (2010). Pathological neovascularization is reduced by inactivation of ADAM17 in endothelial cells but not in pericytes. Circ. Res..

[18] Coleman HA, Labrador JP, Chance RK, Bashaw GJ (2010). The Adam family metalloprotease Kuzbanian regulates the cleavage of the roundabout receptor to control axon repulsion at the midline. Development.

[19] Tabula MC (2018). Single-cell transcriptomics of 20 mouse organs creates a Tabula Muris. Nature.

[20] Seki M, Watanabe A, Enomoto S, Kawamura T, Ito H, Kodama T (2010). Human ROBO1 is cleaved by metalloproteinases and gamma-secretase and migrates to the nucleus in cancer cells. FEBS Lett..

[21] Basagiannis D, Christoforidis S (2016). Constitutive endocytosis of VEGFR2 protects the receptor against shedding. J. Biol. Chem..

[22] Morlot C, Thielens NM, Ravelli RB, Hemrika W, Romijn RA, Gros P (2007). Structural insights into the Slit-Robo complex. Proc. Natl. Acad. Sci. USA..

[23] Chi-Rosso G, Gotwals PJ, Yang J, Ling L, Jiang K, Chao B (1997). Fibronectin type III repeats mediate RGD-independent adhesion and signaling through activated beta1 integrins. J. Biol. Chem..

[24] Swendeman S, Mendelson K, Weskamp G, Horiuchi K, Deutsch U, Scherle P (2008). VEGF-A stimulates ADAM17-dependent shedding of VEGFR2 and crosstalk between VEGFR2 and ERK signaling. Circ. Res..

[25] Basagiannis D, Zografou S, Murphy C, Fotsis T, Morbidelli L, Ziche M (2016). VEGF induces signalling and angiogenesis by directing VEGFR2 internalisation through macropinocytosis. J. Cell. Sci..

[26] Deng Y, Foley EM, Gonzales JC, Gordts PL, Li Y, Esko JD (2012). Shedding of syndecan-1 from human hepatocytes alters very low density lipoprotein clearance. Hepatology.

[27] Galko MJ, Tessier-Lavigne M (2000). Biochemical characterization of netrin-synergizing activity. J. Biol. Chem..

[28] Nishida-Fukuda H, Araki R, Shudou M, Okazaki H, Tomono Y, Nakayama H (2016). Ectodomain shedding of lymphatic vessel endothelial hyaluronan receptor 1 (LYVE-1) is induced by vascular endothelial growth factor A (VEGF-A). J. Biol. Chem..

[29] Li W, Chen Z, Yuan J, Yu Z, Cheng C, Zhao Q (2019). Annexin A2 is a Robo4 ligand that modulates ARF6 activation-associated cerebral trans-endothelial permeability. J. Cereb. Blood Flow. Metab..

[30] Wang L, Fuster M, Sriramarao P, Esko JD (2005). Endothelial heparan sulfate deficiency impairs L-selectin- and chemokine-mediated neutrophil trafficking during inflammatory responses. Nat. Immunol..

[31] Guo C, Fan X, Qiu H, Xiao W, Wang L, Xu B (2015). High-resolution probing heparan sulfate-antithrombin interaction on a single endothelial cell surface: Single-molecule AFM studies. Phys. Chem. Chem. Phys..

[32] Qiu H, Shi S, Yue J, Xin M, Nairn AV, Lin L (2018). A mutant-cell library for systematic analysis of heparan sulfate structure-function relationships. Nat. Methods..

[33] Thieker DF, Xu Y, Chapla D, Nora C, Qiu H, Felix T (2018). Downstream products are potent inhibitors of the heparan sulfate 2-O-sulfotransferase. Sci. Rep..

[34] Moremen KW, Ramiah A, Stuart M, Steel J, Meng L, Forouhar F (2018). Expression system for structural and functional studies of human glycosylation enzymes. Nat. Chem. Biol..

[35] Wang L, Brown JR, Varki A, Esko JD (2002). Heparin's anti-inflammatory effects require glucosamine 6-O-sulfation and are mediated by blockade of L- and P-selectins. J. Clin. Invest..

[36] Song X, Huhle G, Wang L, Hoffmann U, Harenberg J (1999). Generation of anti-hirudin antibodies in heparin-induced thrombocytopenic patients treated with r-hirudin. Circulation.

[37] Song XH, Huhle G, Wang LC, Harenberg J (2000). Quantitative determination of PEG-hirudin in human plasma using a competitive enzyme-linked immunosorbent assay. Thromb. Res..

