# Supplementary material for: Robo4 is constitutively shed by ADAMs from endothelial cells and the shed Robo4 functions to inhibit Slit3-induced angiogenesis
Source: Sci Rep. 2022 Mar 14;12:4352. doi: 10.1038/s41598-022-08227-8 (PMC8921330; doi:10.1038/s41598-022-08227-8)
Supplement: Supplementary file 1 — Supplementary Information. [file 41598_2022_8227_MOESM1_ESM.docx]

**Robo4 is constitutively shed by ADAMs from endothelial cells and the shed Robo4 functions to inhibit Slit3-induced angiogenesis**

Wenyuan Xiao^1,2^, Alejandro Pinilla-Baquero^1^, John Faulkner^1^, Xuehong Song^1^, Pradeep Prabhakar^2^, Hong Qiu^2^, Kelley W. Moremen^2^, Andreas Ludwig^3^, Peter J. Dempsey^4^, Parastoo Azadi^2^, Lianchun Wang^1,2^

^1^ Department of Molecular Pharmacology & Physiology, Byrd Alzheimer`s Research Institute, University of South Florida, 4001 E. Fletcher Ave., Tampa, FL33613, USA

^2^ Complex Carbohydrate Research Center, and Department of Biochemistry and Molecular Biology, University of Georgia, Athens, GA 30602, USA

^3^ Institute for Molecular Pharmacology, RWTH Aachen University, Aachen, Germany

^4^ Department of Pediatrics, University of Colorado Medical School, Aurora, Colorado

Corresponding author: Lianchun Wang, MD

E-mail: [lianchunw@usf.edu](mailto:lianchunw@usf.edu)

**Keywords:** Robo4, shedding, endothelial cell, ADAM, angiogenesis, Slit3, endocytosis

**Supplemental data**

**__Supplemental Figure 1. Mass Spectrometry analysis of the excised 75 kDa protein band determined peptide sequences within the Robo4 ectodomain.** Conditioned medium 6-hour dEC culture was collected, concentrated, and dissolved in SDS-PAGE. The anti-Robo4 ectodomain antibody-positive protein band had a molecular weight of 75 kDa and was excised for MS analysis. The detected peptide sequences were mapped to the mouse protein database in Uniprot. The complete Robo4 sequence was displayed from N- to C-terminus, and the MS-detected unique mouse Robo4 peptides were highlighted in red. Blue, Yellow, and Grey background colors highlight the Ig-, fibronectin type III- and transmembrane domains of mouse Robo4, respectively. All the MS-detected Robo4 peptides were in the Robo4 ectodomain region.

**
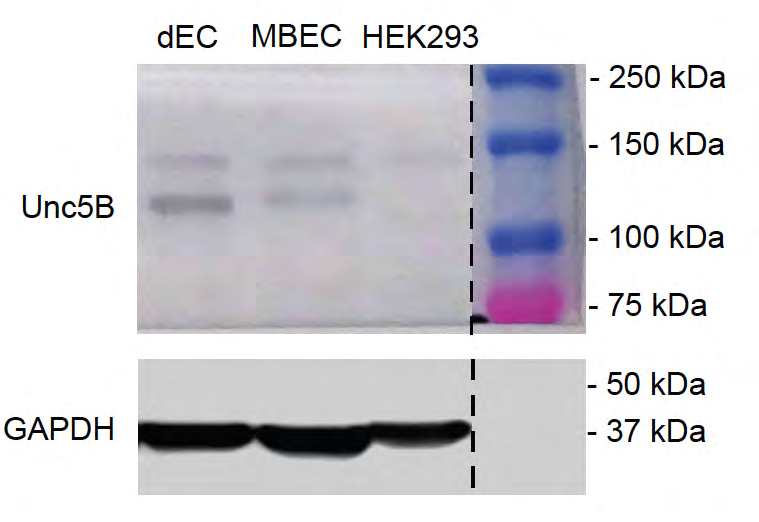
**

**Supplemental Figure 2.** **Unc5B is expressed in dEC and mouse brain endothelial cells (MBEC) but not HEK293 cells.** The dEC, MBEC, and HEK293 cell lysates were probed for Unc5B expression ( 110 kDa band) in Western Blot analysis using a polyclonal anti-Unc5B antibody.

**Supplemental Figure 3. Slit3 does not affect ADAM16 and ADAM17 expression.** After culturing in DMEM for 6 hours, dECs were treated with BSA or Slit3 at1 μg/ml for 6 hours, and expressions of ADAM10 and ADAM17 were examined by Western blot (**A**) and quantified (**B, C**). The ADAM’s signals were normalized to β-actin and then to BSA control. After culturing in DMEM for 6 hours, dECs were treated with BSA or Slit3 at 1 μg/ml in the absence or presence of EIPA at 25 μM for 6 hours. Cell surface ADAM10 (**D**) or ADAM17 (**E**) was assessed by flow cytometry after staining with antibody specific for ectodomain of ADAM10 or ADAM17. The anti-ADAM10/17 staining (heavy-bright lines) was normalized to the naïve IgG controls (thin-faint lines). The data shown represent 3 independent experiments and are presented as mean ± SD. Statistical analysis was carried out using Student`s t-test. ns, not significant.

**Supplemental Figure 4.** **Robo4 shedding was insensitive to inflammatory stimuli.** Treatment of dECs with 0.8 μM PMA or 1 μg/ml LPS for 6 hours in serum-free DMEM induced shedding of Syndecan-1, but not Robo4. The data shown represent 3 independent experiments.

**Original data**

CM shRobo4 CL Total Robo4 CL Actin


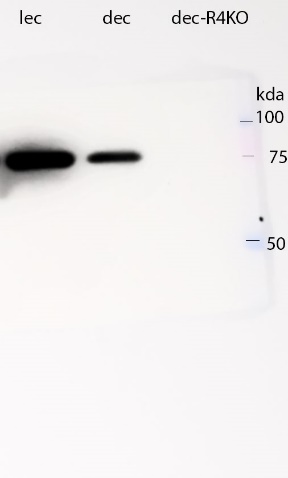

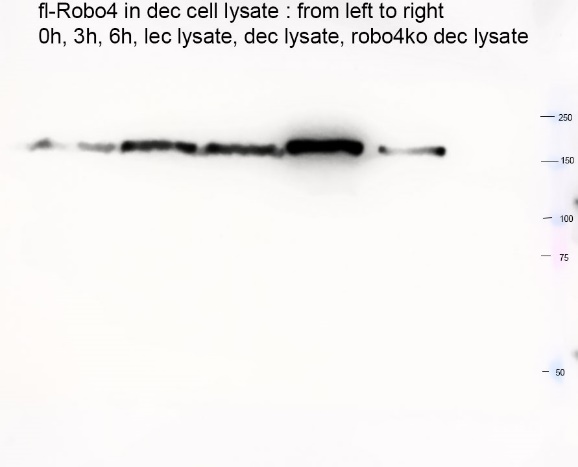

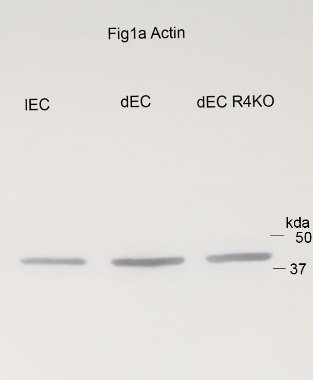


**Uncropped western blot for Figure 1A**. The dotted box demarcates cropped images in Figure 1A. Conditioned media and cell lysates of dECs were collected after the cells were cultured in DMEM for 6 hours. Primary antibodies used are (left-mid: anti-N-Robo4, abcam 10547; right, anti-actin, Sigma A2228). CM = conditioned media; CL = Cell lysate.

Top shRobo4 in CM Total Robo4 in CL Actin in CL


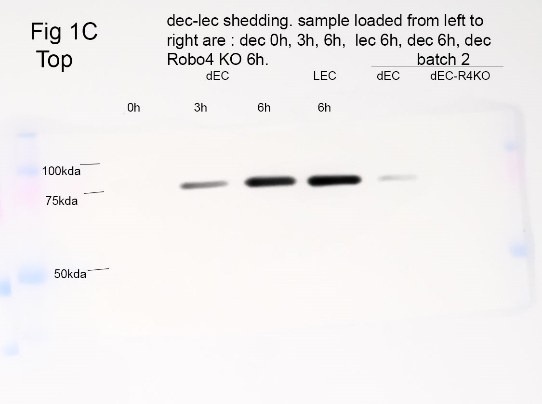

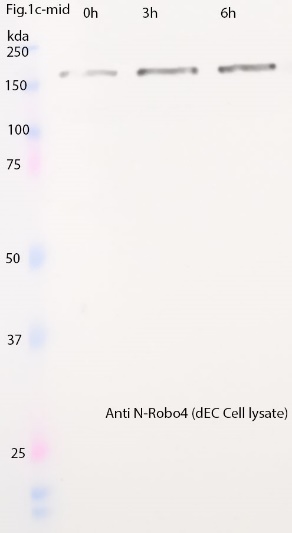

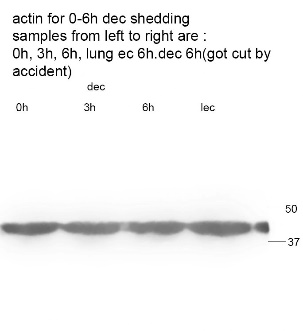


**Uncropped immunoblots for Figure 1C**. The dotted box demarcates cropped images in Figure 1C. Conditioned media and cell lysates of dEC were collected after dEC was cultured in DMEM for 6 hours. Primary antibodies used are (left-mid: anti-N-Robo4, abcam 10547; right, anti-actin, Sigma A2228). CM = conditioned media; CL = Cell lysate)

shRobo4 in CM Total Robo4 in CL


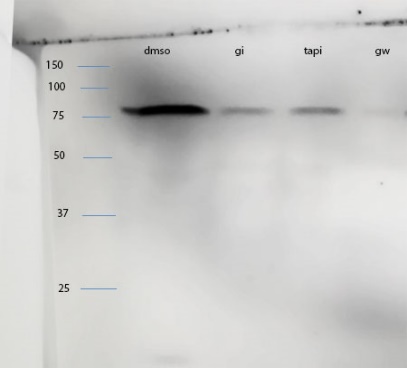

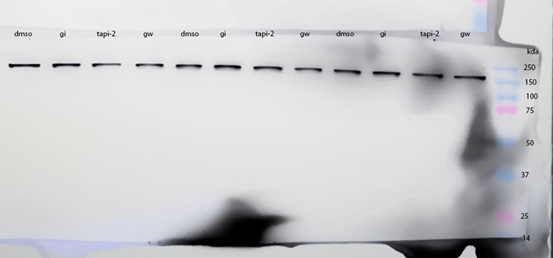


Actin in CL


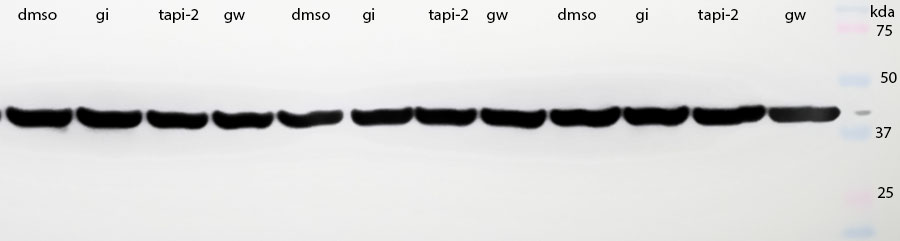


**Uncropped immunoblots for Figure 2C**. The dotted box demarcates cropped images in Figure 2C. Conditioned media and cell lysates of dEC were collected after dEC cultured in DMEM w/o 6 μM corresponding compounds for 6 hours. Primary antibodies used are: top two blots -- anti-N-Robo4, abcam 10547; bottom: anti-actin, Sigma A2228. CM = conditioned media; CL = Cell lysate; GI = GI254023x; GW = GW280264x).

A

_
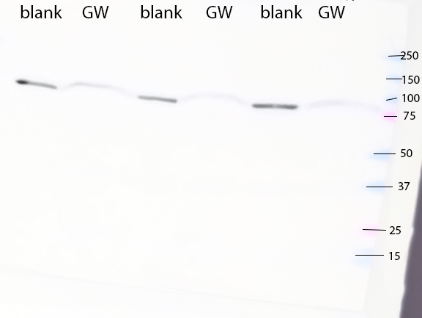
_

B


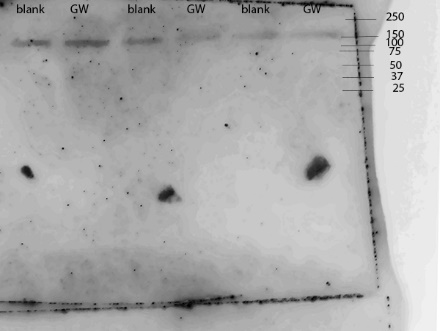


C


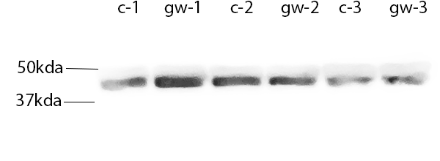


**Uncropped immunoblot for figure 2E.** The dotted box demarcates cropped images in Figure 2E. shRobo4 **(A),** total Robo4 **(B)**, or actin **(C)** in 3 replicates of mouse lung endothelial cells (LECs) were evaluated by western blot. Selected regions from previous figures were cropped in red dashed squares. LECs were treated with 6μM GW208264x or blank control for 6 hours in DMEM at 37 °C. The conditioned media was collected, spun for 10 min at 500g to remove cellular debris, and then concentrated 100x times using 30k protein concentration filters (Merck, UFC803024). Cell lysates were prepared using RIPA buffer (2ml RIPA buffer per plate). The conditioned media and the cell lysates were probed by Rabbit anti-Robo4 ectodomain antibody (Abcam, ab10547) followed by Goat-anti Rabbit secondary antibody (Santa Cruz, sc-2030) in western blot. Actin was probed by beta-actin monoclonal antibody-HRP (Fisher Scientific, HRP-60008). Bound HRP was exposed by ECL substrate (Kwikquant R1002). The results have been repeated more than once.

A

_
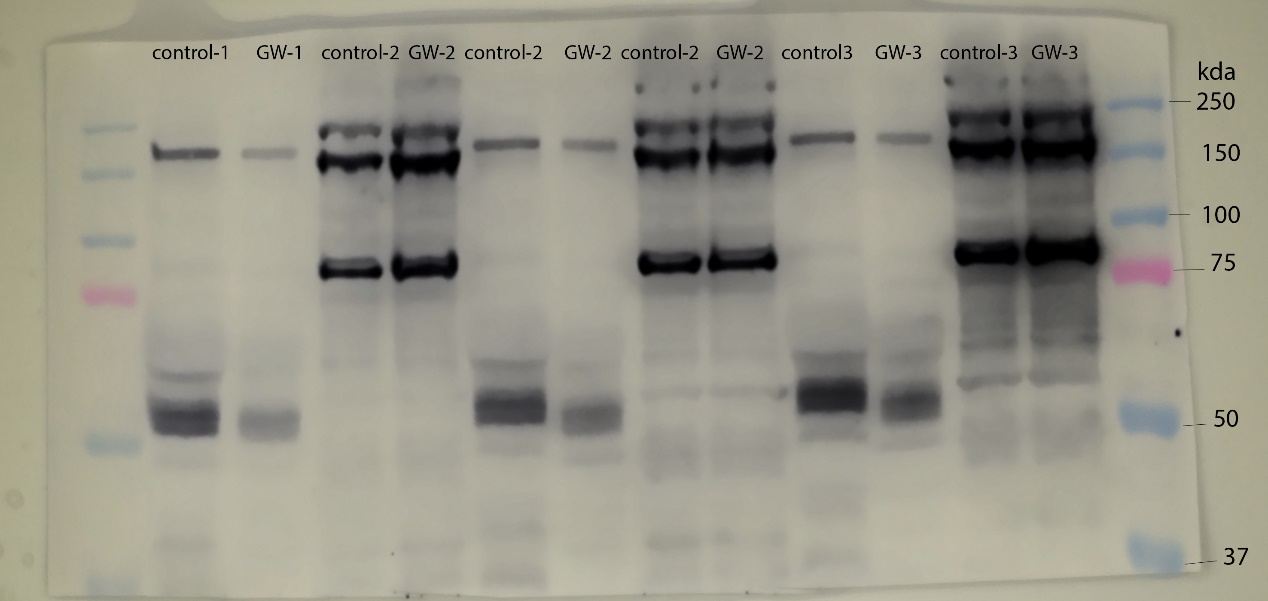
_

B

_
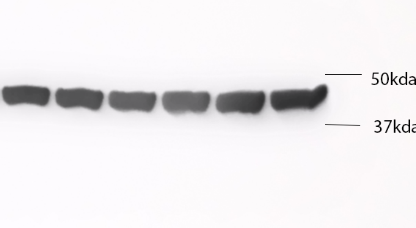
_

**Uncropped immunoblot for figure 2F**. The dotted box demarcates cropped images in Figure 2F. shRobo4/total Robo4 **(A)** and actin **(B)** in 3 replicates of P6 HUVEC were evaluated by western blot. Selected regions from previous figures were cropped in red or blue dashed squares. P6 HUVEC were treated with 6μM GW208264x or blank control for 6 hours in DMEM at 37 degrees. The conditioned media was collected, spun for 10 min at 500g to remove cellular debris, and then concentrated 100x times using 30k protein concentration filters (Merck, UFC803024). Cell lysates were prepared using RIPA buffer (2ml RIPA buffer per plate). The conditioned media and the cell lysates were probed by Rabbit anti-Robo4 ectodomain antibody (Abcam, ab10547) followed by Goat-anti Rabbit secondary antibody (Santa Cruz, sc-2030) in western blot. Actin was probed by beta-actin monoclonal antibody-HRP (Fisher Scientific, HRP-60008). Bound HRP was exposed by ECL substrate (Kwikquant R1002). The results were repeated twice.

ADAM10 in cell lysate ADAM17 in cell lysate Actin in cell lysate


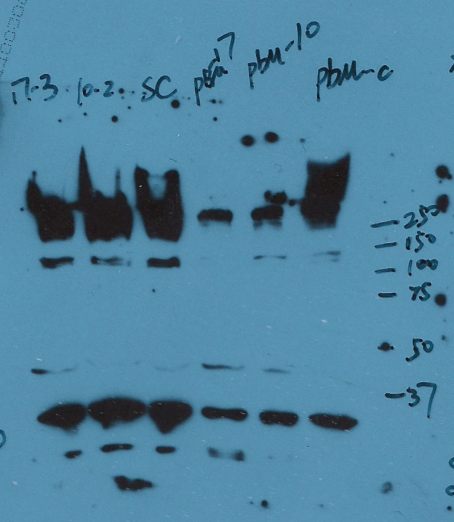

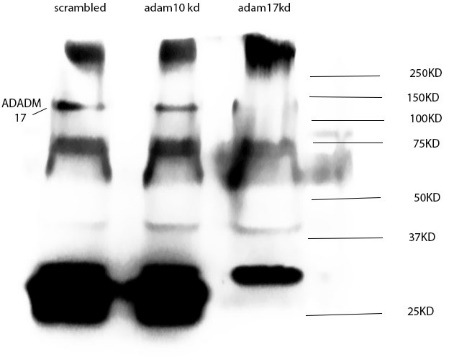

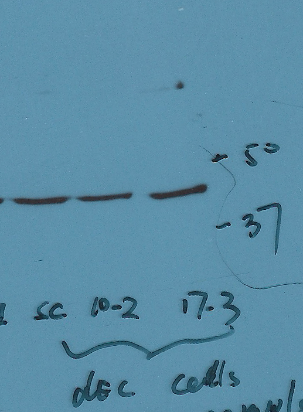


**Uncropped immunoblots for Figure 2G**. The dotted box demarcates cropped images in Figure 2G. Conditioned media and cell lysates of dEC were collected after dEc cultured in DMEM w/o corresponding compounds for 6 hours. Primary antibodies used are (anti ADAM10, Bioss bs-3574R; anti ADAM17, Bioss 4236R; anti-actin, Sigma A2228). SC = scrambled control; 10-2 = the second plasmid of the commercial shRNA plasmid sets of ADAM10 knockdown (KD); 17-3 = the third plasmid of the commercial shRNA plasmid sets of ADAM17 KD; pbm-c/pbm-10/pbm-17 are datasets not reporting in this article).

Top shRobo4 in CM Total Robo4 in CL Actin in CL


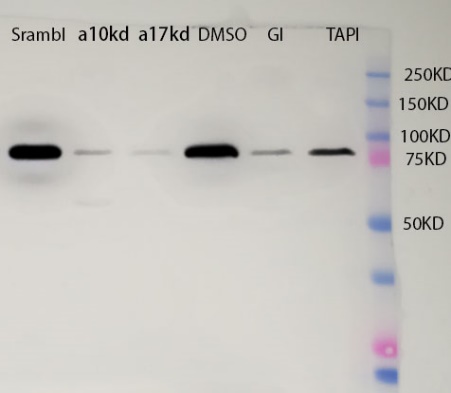

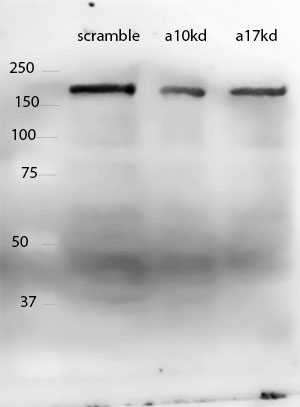

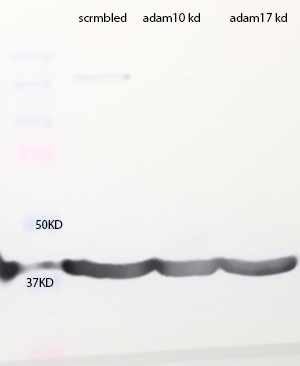


**Uncropped immunoblots for Figure 2H**. The dotted box demarcates cropped images in Figure 2H. Conditioned media and cell lysates of dEC were collected after dEC was cultured in DMEM for 6 hours. Primary antibodies used are (left-mid: anti-N-Robo4, abcam 10547; right, anti-actin, Sigma A2228). CM = conditioned media; CL = Cell lysate.

OE hRobo4 in CL Actin in CL


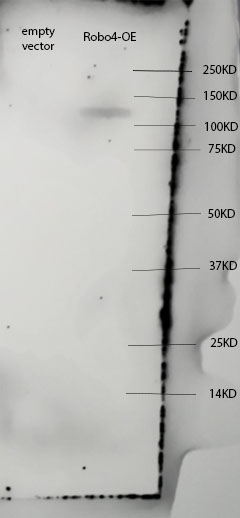

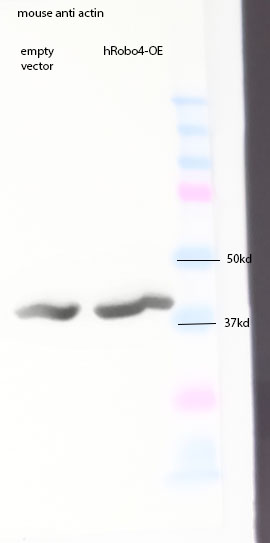


**Uncropped immunoblots for Figure 3A**. The dotted box demarcates cropped images in Figure 3A. Cell lysates were collected from dEC transiently expressing blank or hRobo4-HA-FLAG. Primary antibodies used are (left, R&D MAB2524; right, anti-actin, Sigma A2228).

hRobo4-HA-FLAG in cell lysates Actin in cell lysates


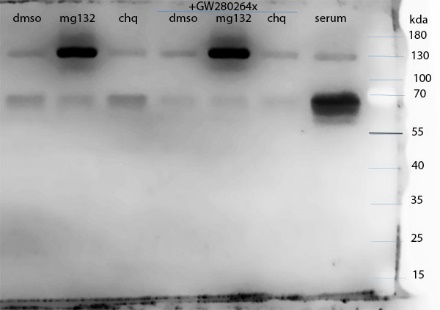

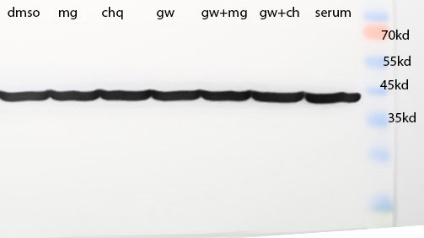


**Uncropped immunoblots for Figure 3B**. The dotted box demarcates cropped images in Figure 3B. After starvation for 3 hours in DMEM and treatment with corresponding reagent (6 μM GW280264x; 12μM Mg132; 50μM Chloroquine diphosphate-chq) for 6 hours, cell lysates were collected from dEC transiently expressing hRobo4-HA-FLAG. Primary antibodies used are (left, anti-FLAG, Thermo Fisher, 14-6681-82; right, anti-actin, Sigma A2228). Note: serum treatment robustly increased Robo4 c-terminal fragment).

hRobo4-HA-FLAG in cell lysate Actin in cell lysate


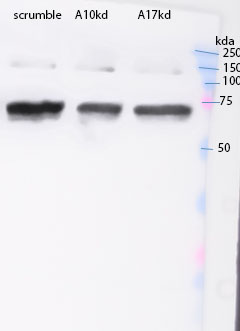

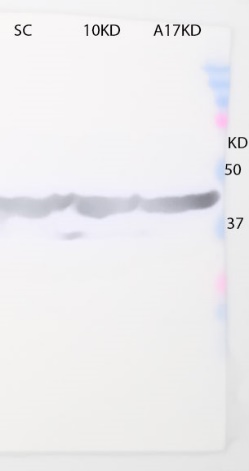


**Uncropped immunoblots for Figure 3C.** The dotted box demarcates cropped images in Figure. After starvation for 6 hours in DMEM, cell lysates were collected from dEC transiently expressing hRobo4-HA-FLAG in scrambled control, ADAM10 knock-down, or ADAM17 knock-down background. Primary antibodies used are (left, anti-FLAG, Thermo Fisher, 14-6681-82; right, anti-actin, Sigma A2228).

Left: Robo4 in cell lysate Actin in cell lysate


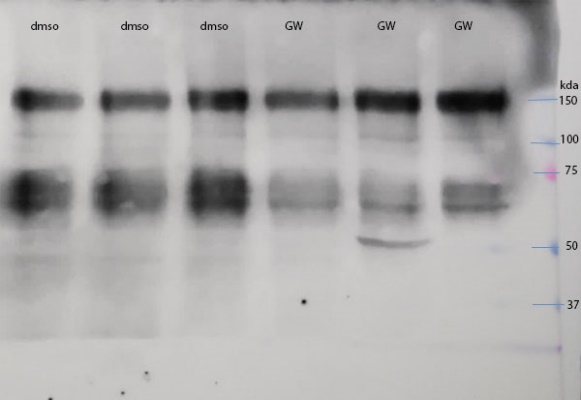

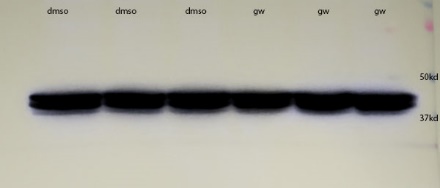


Right: Robo4 in cell lysate Actin in cell lysate


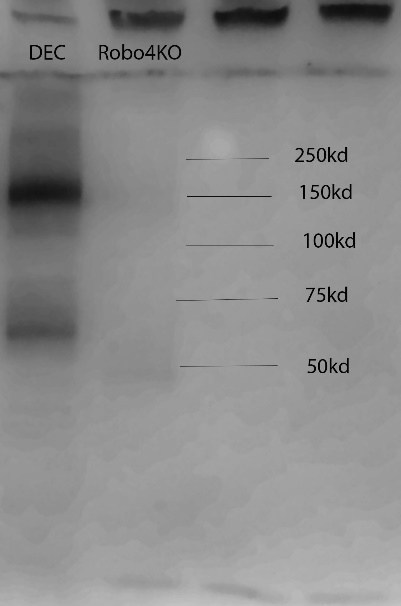

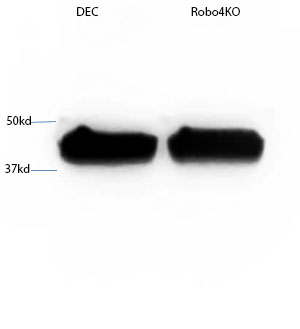


**Uncropped immunoblots for Figure 3D**. The dotted box demarcates cropped images in Figure. **TOP two blot**s: Treatment with DMSO or 6 μM GW280264x (GW) for 6 hours, dEC cell lysates were prepared and probed with an antibody against endogenous mouse Robo4 c-terminal fragment (Santa cruz, SC-46497) or Actin (Sigma, A2228). Bottom two blots: verification of the sc-46497 antibody in dEC or dEC with Robo4 KO genotype.

Cdh5 in conditioned media and cell lysates Actin in cell lysates


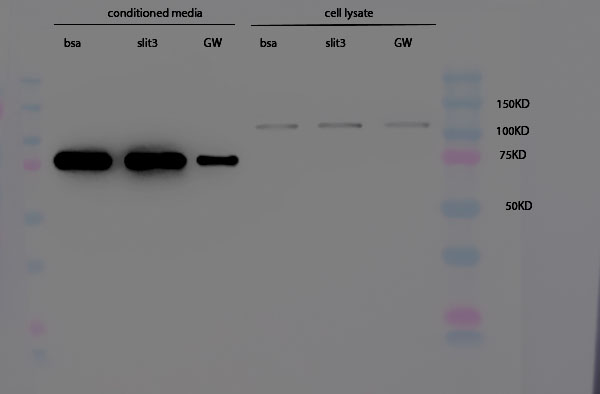

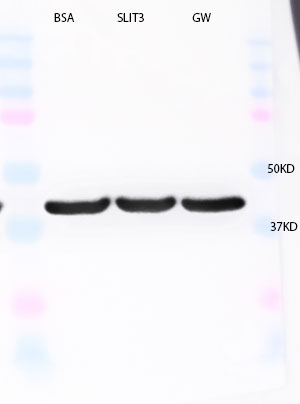


Uncropped immunoblots for Figure 5A. The dotted box demarcates cropped images in Figure 5A. dECs were starved for 6 hours in DMEM and treated with 1 μg/ml BSA or Slit3 or 6 μM GW280264x (GW). Conditioned media and cell lysates were collected and blotted with a primary antibody against Cdh5 (Fisher scientific, 14-1441-82) or Actin (Sigma, A2228).

A

_
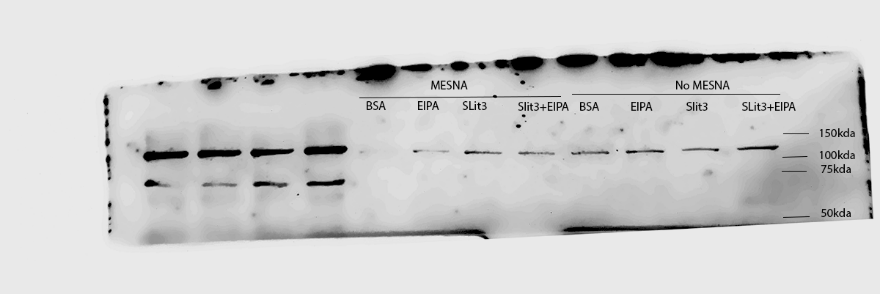
_

B

_
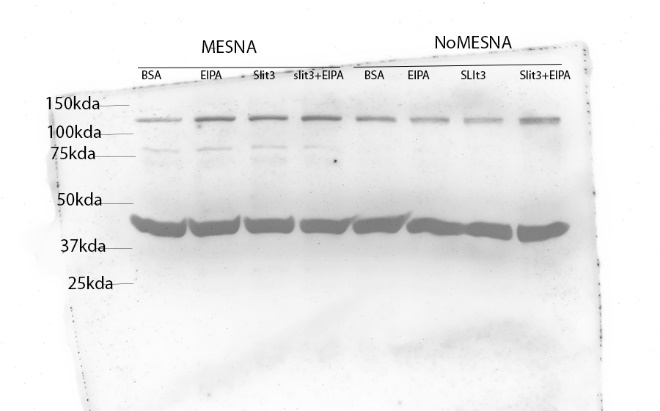
_

C


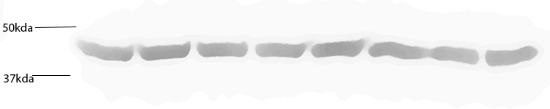


**Uncropped immunoblot for Figure 5C**. The dotted box demarcates cropped images in Figure 5C. Internalized cell surface hRobo4-FLAG (MESNA group, **A**), total extracellularly biotinylated hRobo4-FLAG (No. MESNA group, **A**), total hRobo4-FLAG (B), and actin (**C**) were probed by beta-actin monoclonal antibody-HRP (Fisher Scientific, HRP-60008) or Anti-FLAG antibody (Thermo Fisher, #14-6681-82)/HRP-conjugated goat anti-mouse IgG (Novus Biologicals, NBP1-75130) in 1x TBST (4% milk) in western blot. In detail, 90% of confluent dECs seeded in a 150cm cell culture plate were starved for 7 hours in DMEM. Then cell surface proteins were biotinylated with 0.25mg/ml Sulfo-NHS-SS-biotin in 1x DPBS (ph=7.4) at 4 °C for one hour following commercial protocol (Thermo Fisher #21331). Then cells were treated with DMSO or 25μM EIPA in DMEM for 10 min with 1μg/ml BSA or Slit3 for 10 min at room temperature and then for another 30 min in cell culture incubator (37 °C with 5% CO). Then cells were lysed with 2ml/plate RIPA buffer directly or incubated with ice-cold 100mM MESNA (Fisher Scientific, 50-163-8016) in DPBS (ph=7.4) twice, 15 min each time to remove cell surface biotin. After four additional washes with ice-cold DPBS, cells were lysed with 2ml/plate RIPA buffer. Biotinylated protein was pulled down from cell lysates using 50ul resuspended Neutravidin agarose (Thermo fisher #29201) at 4 °C overnight and then were probed by Anti-FLAG antibody (Thermo Fischer #14-6681-82) and HRP-conjugated goat anti-mouse IgG antibody (Novus Biologicals, NBP1-75130) in 1x TBST (4% milk) in western blot **(A)**. Blots containing total cell lysate samples were first incubated with anti-FLAG antibody overnight in 1x TBST (4% milk) at 4 °C, followed by incubation with HRP-conjugated goat anti-mouse IgG antibody (1x TBST, 4% milk) for 2 hours at room temperature. **(B)**. Then antibodies were washed 3 times with 1xTBST and incubated with beta-actin monoclonal antibody-HRP (Fisher Scientific, HRP-60008) for another half an hour in 1x TBST-4% milk at room temperature. Bound HRP was exposed by ECL substrate (Kwikquant R1002). Images used in previous figures were cropped in dashed red squares. The arrow shows actin in the total cell lysate. The result has been repeated more than once.

shRobo4 in the conditioned media


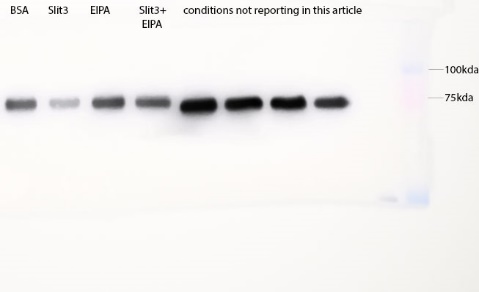


Robo4 in cell lysate Actin in cell lysate


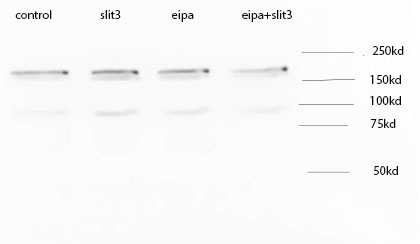

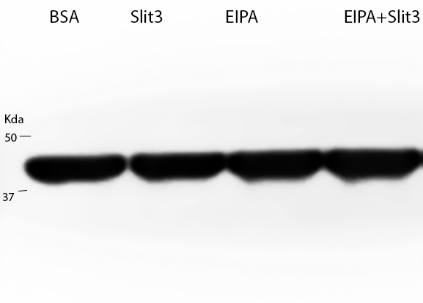


**Uncropped immunoblots for Figure 5D**. The dotted box demarcates cropped images in Figure 5D. DEC was starved for 6hours in DMEM and treated with 1 μg/ml BSA or Slit3 w/o 25 μM EIPA. Conditioned media (top) and cell lysates (bottom) were collected and blotted with a primary antibody against Robo4 (Abcam, 10547) or Actin (Sigma, A2228).

hRobo4-HA-FLAG in cell lysate Actin in cell lysate


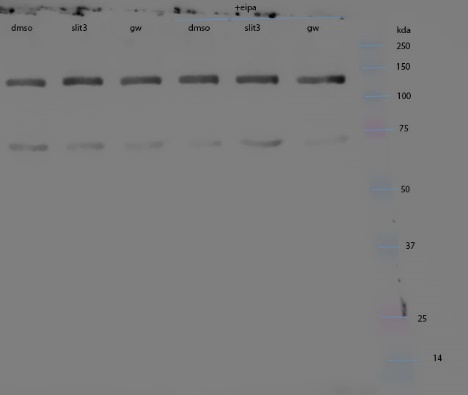

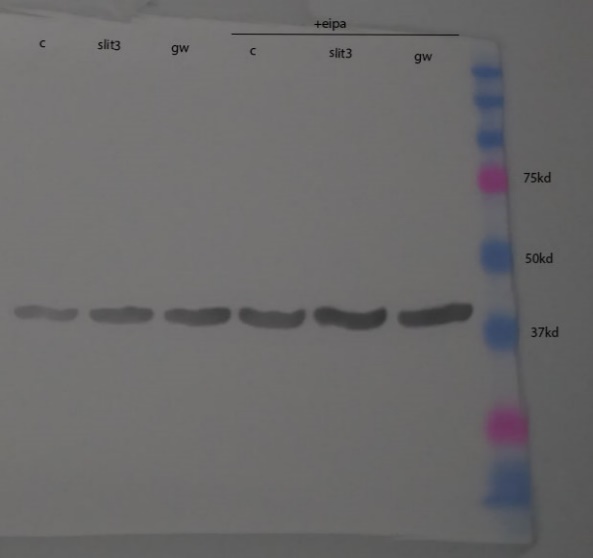


**Uncropped immunoblots for Figure 5E.** The dotted box demarcates cropped images in Figure 5E. dECs were starved for 6 hours in DMEM and treated with 1 μg/ml BSA or Slit3 w/o 6 μM GW280264x or 25 μM EIPA. Cell lysates were collected and blotted with primary antibody against FLAG (Thermo Fisher, 14-6681-82) or Actin (Sigma, A2228).


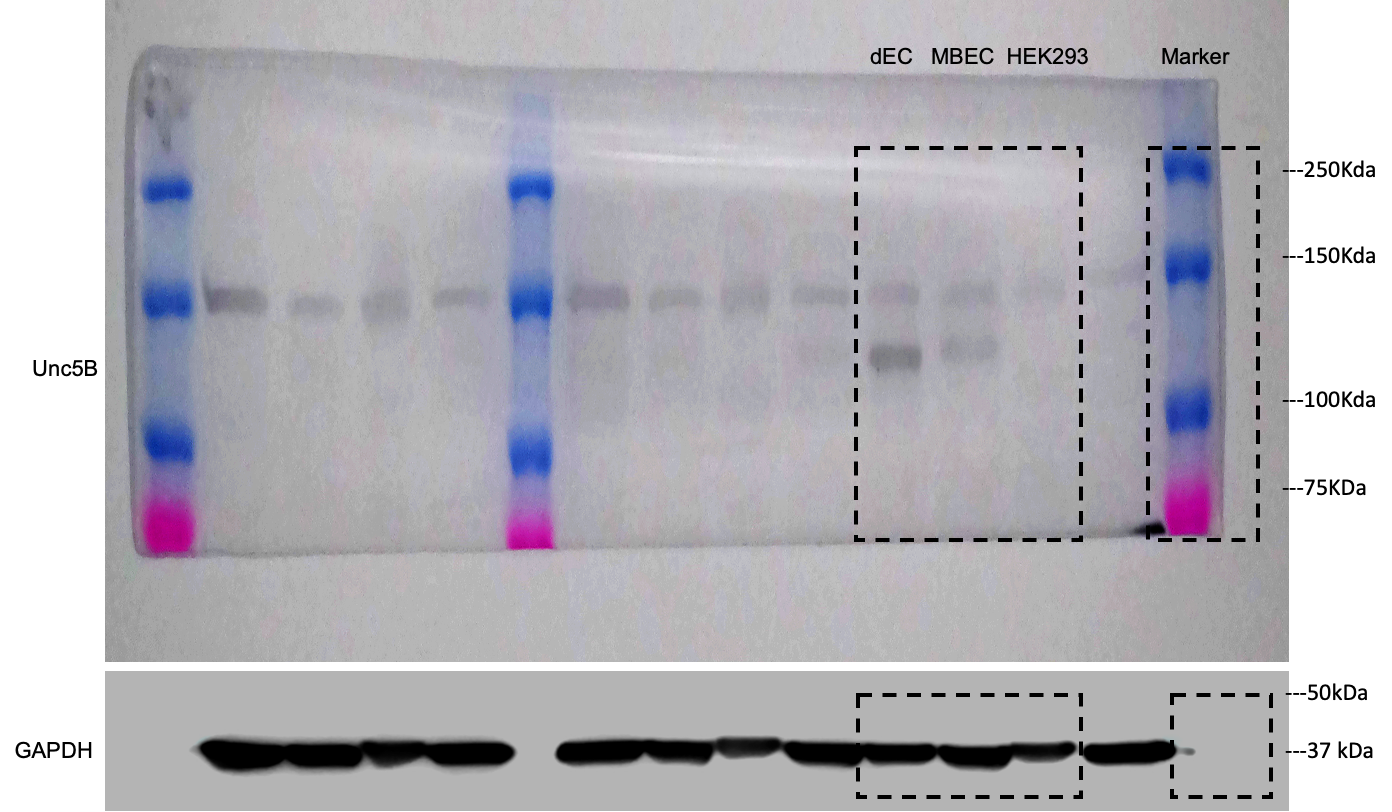


**Uncropped immunoblots for supplemental Figure 2.** The dotted box demarcates cropped images in the supplementary Figure 2. The cell lysates (37.5 μg/lane) of dEC, MBEC (mouse brain endothelial cells), and HEK293 were separated in 7.5% SDS-PAGE, and then transferred to polyvinylidene difluoride membrane. After blocking, the membrane was probed with a polyclonal anti-Unc5B antibody at 1 µg/ml and followed by an HRP-conjugated secondary antibody at 300 ng/ml. Probed Unc5B was detected via chemiluminescence using the Kwik quant imager system (Kindle Biosciences, D1001).

Adam10 in cell lysate ADAM17 in cell lysate Actin in cell lysate


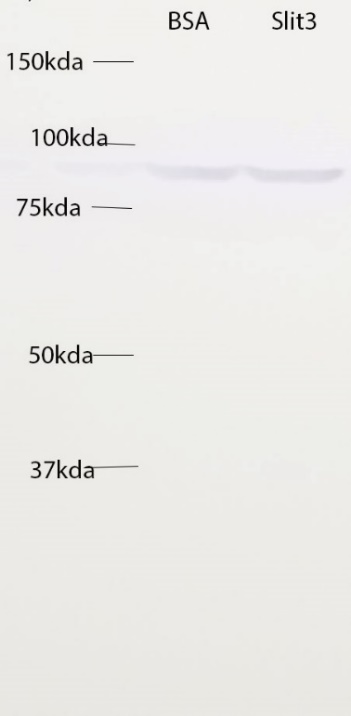

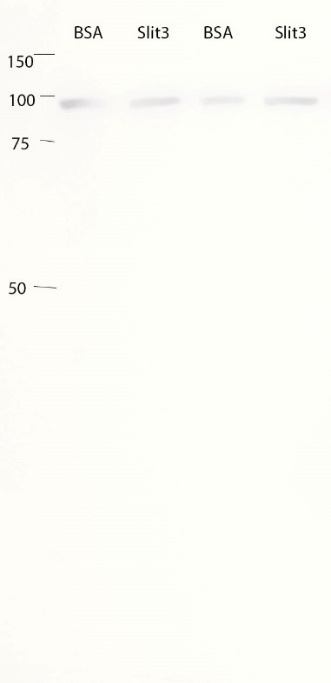

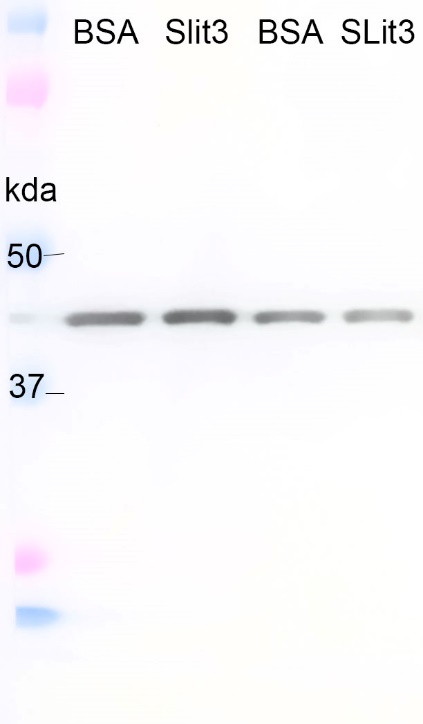


**Uncropped immunoblots for supplemental Figure 3.** The dotted box demarcates cropped images in the supplementary Figure 3. dECs were starved for 6hours in DMEM and then treated with 1μg/ml BSA or Slit3 for another 6 hours. Cell lysates were collected and blotted with a primary antibody against Adam10 (Bioss, bs-3574R), ADAM17 (Bioss, 4236R), or Actin (Sigma, A2228). Note: the molecular weight of ADAM10 and ADAM17 is smaller than those in Figure 2D and Supplementary Figure 7. We believe the previous larger ADAM10/17 are pro-proteins, and smaller ADAM10/17 in this figure are mature ADAM10/17 whose pro-domain has been removed.

PMA: shRobo4 in CM Robo4 in CL Actin in CL


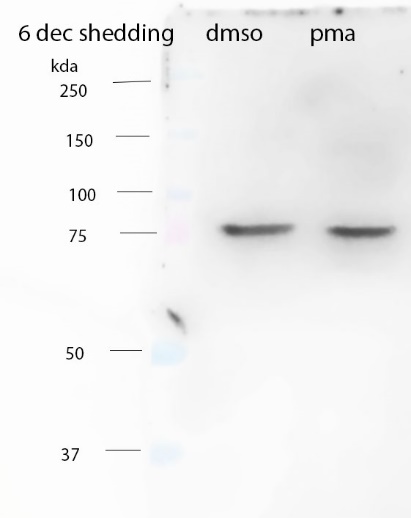

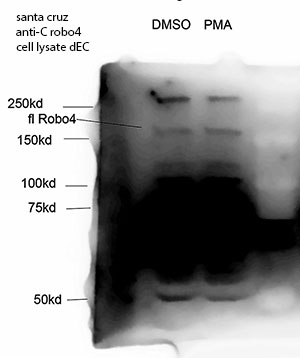

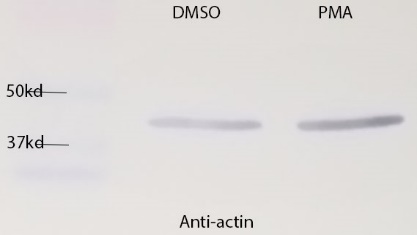


LPS: sRobo4 in CM Robo4 in CL Actin in CL


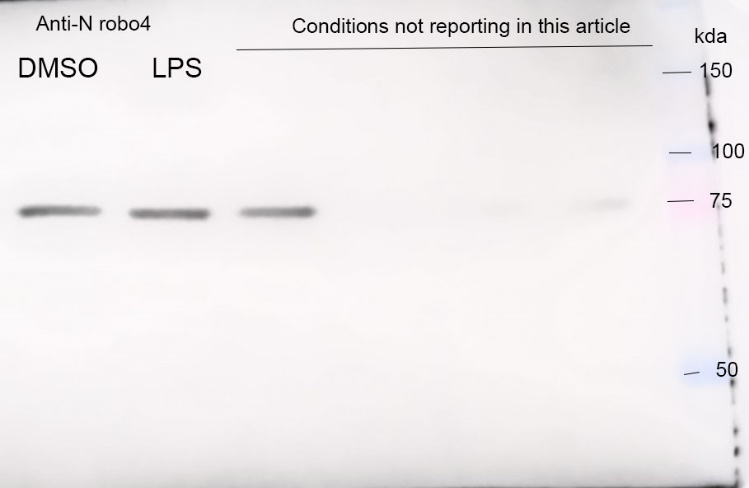

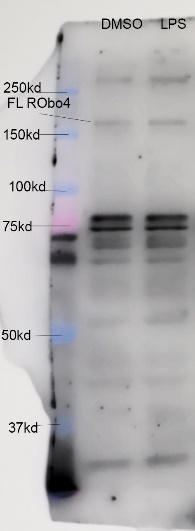

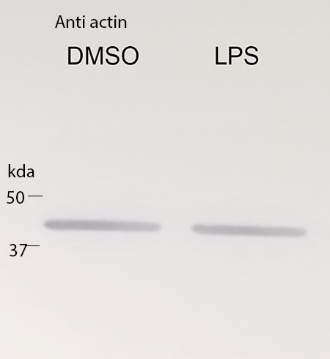


Syndecan-1 in CM Syndecan-1 in CL Actin in CL


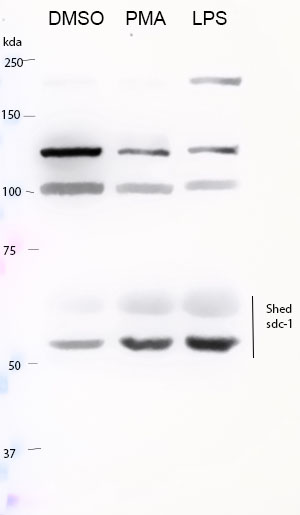

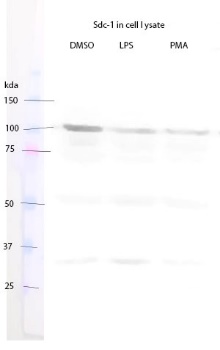

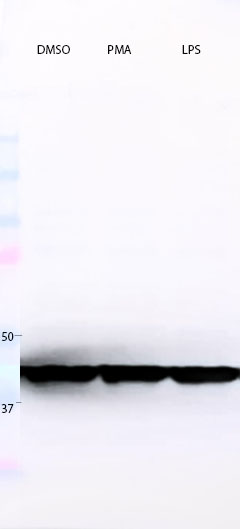


**Uncropped immunoblots for supplemental Figure 4.** The dotted box demarcates cropped images in Supplementary Figure 4. **Top 3 blots**: DEC was treated with DMSO or 0.8μM phorbol 12-myristate 13-acetate (PMA) for 6 hours in DMEM. shRobo4 was then detected through primary antibodies against N-terminal Robo4 (Abcam, 10547). Cell lysates were collected and blotted with primary antibody against the c-terminal of Robo4 (Santa cruz, sc-46497) or Actin (Sigma, A2228). **Middle 3 blots**: DEC was treated with DMSO or 1μg lipopolysaccharide (LPS) for 6 hours in DMEM. shRobo4 was then detected through primary antibody against N-terminal Robo4 (Abcam, 10547). Cell lysates were collected and blotted with primary antibodies against the c-terminal of Robo4 (Santa cruz, sc-46497) or Actin (Sigma, A2228). **Bottom 3 blots**: dECs were treated with DMSO or 0.8 μM phorbol 12-myristate 13-acetate (PMA) or 1 μg lipopolysaccharide (LPS) for 6 hours in DMEM. Shed Syndecan-1 was then detected through primary antibody against N-terminal Robo4 (Santa cruz, sc-5632). Cell lysates were collected and blotted with primary antibodies against Syndecan-1 (Santa cruz, sc-5632) or Actin (Sigma, A2228).

**Supplementary Data-1:** The peptide sequences in the anti-Robo4 ectodomain antibody satined 75 kDa protein band detected in proteomics analysis.
